# Molecular basis of IFN-γ–induced STAT3 phosphorylation stimulated by Sendai virus C protein

**DOI:** 10.1016/j.jbc.2025.110744

**Published:** 2025-09-18

**Authors:** Kosuke Oda, Yuta Hatori, Atsuji Kodama, Susumu Uchiyama, Takashi Oda, Yasuyuki Matoba, Ami Nakano, Kanako Ninomiya, Seira Yoshidomi, Takemasa Sakaguchi

**Affiliations:** 1 Faculty of Pharmacy, Yasuda Women’s University, Hiroshima, Japan; 2 Department of Virology, Institute of Biomedical and Health Sciences, Hiroshima University, Hiroshima, Japan; 3 Exploratory Research Center on Life and Living Systems (ExCELLS), National Institutes of Natural Sciences, Okazaki, Japan; 4 Department of Biotechnology, Graduate School of Engineering, Osaka University, Osaka, Japan; 5 Department of Life Science, Rikkyo University, Tokyo, Japan

**Keywords:** C protein, interferon, negative-strand RNA virus, paramyxovirus, protein–protein interaction, Sendai virus, signal transduction, STAT3

## Abstract

Sendai virus, belonging to the *Respirovirus* genus in the *Paramyxoviridae* family, possesses C protein to escape from host innate immunity by inhibiting the IFN-α/β–induced STAT1:STAT2 pathway and the interferon (IFN)-γ–induced STAT1 pathway *via* binding to the *N*-terminal domain of STAT1 (STAT1ND). In this study, a yeast two-hybrid analysis revealed that C protein also binds directly to the *N*-terminal domain of STAT3 (STAT3ND). The *C*-terminal region of C protein (named Y3) was sufficient for binding to STAT3ND, similar to STAT1ND binding. However, the affinity of Y3 for STAT3ND was significantly weaker than that for STAT1ND. Transfection experiments using 293T cells demonstrated that the introduction of C protein significantly stimulated the IFN-γ–induced phosphorylation of STAT3. Considering the results of stoichiometric and confocal analyses together, C protein likely plays a role in stabilizing the dimeric structure formed by STAT3ND, stimulating the recruitment of dimeric STAT3 to the plasma membrane. Reporter assay demonstrated the persistent activation of the STAT3 pathway in the presence of C protein after IFN-γ stimulation. The STAT1 homodimer, bound to two molecules of C protein, cannot take an active form to promote the transcription of target genes. In contrast, STAT3 can take an active form even in the presence of C protein, probably because the complex formed between them is fragile.

Sendai virus (SeV), a member of the *Respirovirus* genus in the *Paramyxoviridae* family, causes acute respiratory infection in rodents. Extensive research has provided insights into the molecular and biological properties of human parainfluenza virus type 1 (hPIV1) and hPIV3, which are responsible for respiratory illnesses in young children and can lead to occasional outbreaks in immunocompromised and older patients (1, 2, 3). Understanding SeV is instrumental for the development of effective antiviral treatments against hPIVs.

SeV produces C proteins, which are translated from P and V mRNAs in a coding frame differing from that of P and V proteins. C proteins consist of C (aa 1–204), Y1 (aa 24–204), Y2 (aa 30–204), and C′ (which has an additional 11 aa at the *N* terminus of C), which have different initiated *N* terminus and common *C* terminus, respectively (Fig. 1*A*). Among C proteins, C is the main protein expressed in infected cells (4, 5). C proteins play a crucial role in viral replication *in vitro* and are essential for the multiplication and pathogenicity of viral infection in living organisms (6). The fact that revertant SeVs is rapidly generated from recombinant SeVs expressing none of the C proteins during serial passages in embryonated chicken eggs further emphasizes the pivotal role of C protein (7).

**Figure 1:**
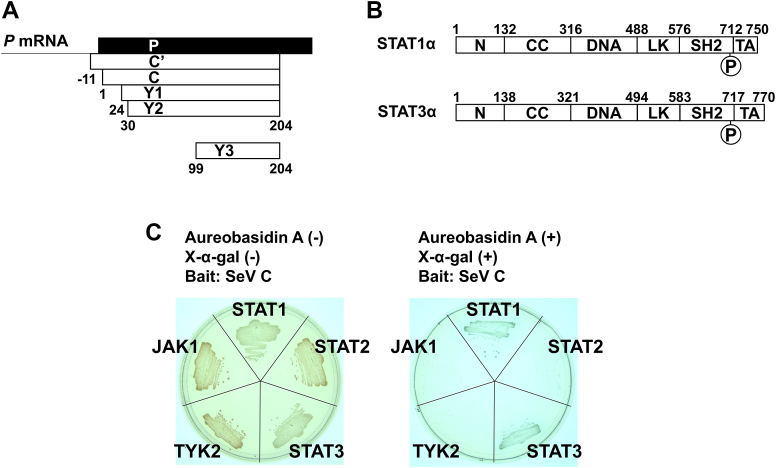
**Yeast two-hybrid analysis using SeV C protein and IFN-α–related proteins.***A*, schematic diagrams of C′, C, Y1, Y2, and Y3. *B*, linear representations of the domains in human STAT1α and STAT3α. P indicates phosphorylation at the Tyr 701 residue for STAT1 and at the Tyr 705 residue for STAT3. *C*, yeast two-hybrid analysis using SeV C protein as bait and IFN-α–related proteins as prey. Experiments were conducted in the absence or presence of aureobasidin A and X-α-gal for colony selection. When a bait protein bound to a prey protein, the *MEL1* gene was expressed, hydrolyzing X-α-gal into a *blue* pigment. N, *N*-terminal domain; CC, coiled-coil domain; DNA, DNA-binding domain, JAK1, Janus kinase 1; LK, linker domain; SH2, SH2 domain; TA, transcription activation domain, TYK2, Tyrosine kinase 2; SeV, Sendai virus; IFN, interferon; STAT, signal transducer and activator of transcription.

C protein inhibits the antiviral effects of interferon (IFN)-α/β and IFN-γ by binding to the signal transducer and activator of transcription 1 (STAT1) (8, 9, 10, 11, 12, 13). The *N*-terminal domain of STAT1 (STAT1ND) is responsible for the dimerization of the nonphosphorylated form (14, 15, 16). STAT1ND links to the core fragment, including a DNA-binding domain, SH2 domain, and tyrosine residue targeted for phosphorylation *via* a flexible linker peptide (Fig. 1*B*), leading to the structural diversity of the STAT1 homodimer (17). The *C*-terminal region of C protein (named Y3) binds to STAT1ND (12) (Fig. 1, *A* and *B*). In a previous report, we proposed a mechanism for the inhibition of the STAT1 pathway by C protein based on the crystal structure of the Y3:STAT1ND complex (12).

C protein also facilitates the budding of SeV from infected cells by recruiting the host ALG-2–interacting protein X (Alix), a component of the endosomal sorting complexes required for the transport machinery, to the cellular membrane (18, 19). In addition, C protein regulates viral RNA synthesis to reduce abnormal RNA species (20, 21, 22, 23) and control viral genome polarity (24, 25) by interacting with the L protein, an RNA-dependent RNA polymerase. Recently, C protein was found to inhibit macrophage functions to cause phagocytosis and produce nitric oxide, thereby exaggerating pulmonary inflammation and disease in SeV-infected mice (26, 27, 28).

The molecular mechanisms related to the action of C protein, which have been partially explained by the binding of C protein to the well-known targets STAT1 and Alix, can be elucidated by identifying a novel interaction partner. The present study discovered that C protein directly binds to STAT3, another transcription factor in the IFN-γ signaling pathway. The interaction leads to the increased activation of the STAT3 pathway in response to IFN-γ stimulation. Additionally, we discuss the molecular mechanism whereby C protein stimulates the IFN-γ–induced phosphorylation of STAT3. These findings not only contribute to uncovering the potential role of C protein in the viral life cycle but also have significant implications for developing antiviral agents by targeting the STAT1 and STAT3 pathways in IFN-γ signaling.

## Results

### Binding of C protein to STAT3

C protein is known to inhibit the IFN-α–induced phosphorylation of STAT2 *via* the binding to STAT1ND (13). However, the protein also inhibits the phosphorylation of STAT2 in an unknown STAT1-independent manner (13). C protein is also known to inhibit the phosphorylation of two nonreceptor tyrosine kinases, Janus kinase 1 (JAK1) and tyrosine kinase 2 (TYK2), in a currently unknown mechanism (29). These motivated us to conduct a yeast two-hybrid assay using SeV C protein as bait and IFN-α–related factors STAT2, STAT3, JAK1, and TYK2 as prey to identify a novel binding target of C protein. When using STAT3 as prey, blue colonies were grown as well as when using STAT1 as a positive control of the prey (Fig. 1*C*). The interaction between C protein and STAT3 was not confirmed as a false-positive reaction (Fig. S1). In contrast, no interaction of C protein with STAT2, JAK1, or TYK2 was detected (Fig. 1*C*).

**Figure 2:**
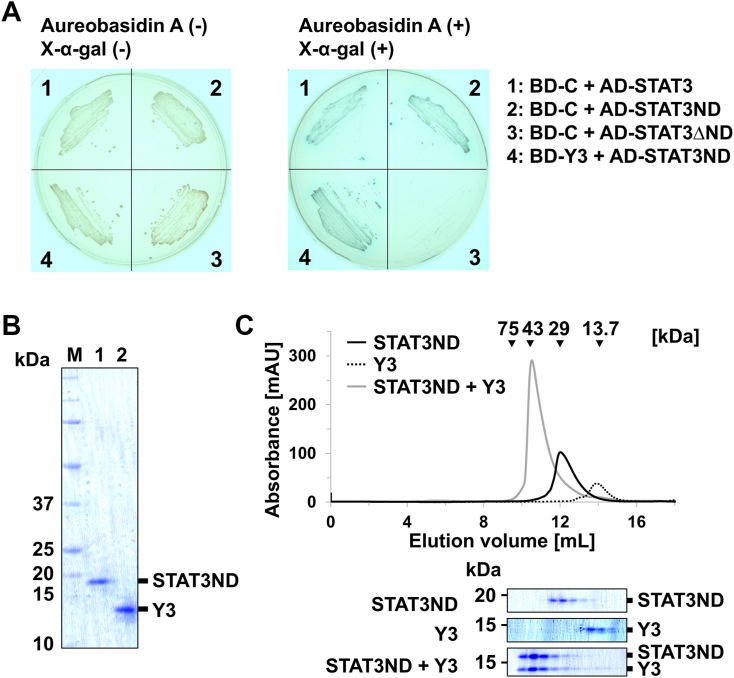
**Identification of the binding region between C protein and STAT3.***A*, yeast two-hybrid analysis using C protein or Y3 as bait and STAT3, STAT3ND, or STAT3ΔND as prey. Experiments were performed in the absence or presence of aureobasidin A and X-α-gal for colony selection. *B*, SDS-PAGE analysis of STAT3ND and Y3 used for SEC analysis. *C*, SEC analysis of STAT3ND in the absence (*black line*) or presence (*gray line*) of Y3. STAT3ND (50 μM) was preincubated with or without an equimolar concentration of Y3 for 10 min, followed by SEC analysis. A chromatogram of Y3 alone is shown as a *dashed line*. Proteins in the fractions eluted from the size-exclusion chromatograms were separated by SDS-PAGE, followed by Coomassie *blue* staining. STAT, signal transducer and activator of transcription; STAT3ND, *N*-terminal domain of STAT3; SEC, size-exclusion chromatography.

STAT3 comprises an *N*-terminal domain and core fragment similar to STAT1 (Fig. 1*B*) (17). A yeast two-hybrid assay was also conducted to determine the domains essential for complex formation between C protein and STAT3. The results revealed that C protein targets the *N*-terminal domain of STAT3 (STAT3ND) but not the core fragment (Fig. 2*A*). Furthermore, Y3 was found to contain a region sufficient for binding to STAT3ND (Fig. 2*A*). To further investigate this interaction, Y3 and STAT3ND were prepared using an *Escherichia coli* expression system (Fig. 2*B*). Size-exclusion chromatography (SEC) was performed to confirm the interaction between Y3 and STAT3ND. The calculated molecular masses of monomeric Y3 and STAT3ND are approximately 14.5 kDa and 17 kDa, respectively. When using the protein solution with a concentration adjusted to 100 μM, Y3 and STAT3ND eluted at approximately 14 ml and 12 ml of the elution volume, corresponding to 14 kDa and 29 kDa, respectively (Fig. 2*C*). Thus, STAT3ND may have a homodimeric structure, while Y3 may have a monomeric structure. When the mixed solution was used, Y3 coeluted with STAT3ND at approximately 10.5 ml, corresponding to 51 kDa. These results underscore that C protein binds to STAT3 through its interaction with the *N*-terminal domain.

### Stoichiometric analysis of the Y3:STAT3ND complex

A mixed solution of Y3 and STAT3ND, containing both proteins at the same molar concentration, was prepared to determine the stoichiometry of the Y3:STAT3ND complex (Fig. S2*A*). When the concentrations were set to 8 μM, SEC with multiangle light scattering (MALS) analysis estimated that the elution peak has a molecular mass of approximately 23 kDa, suggesting that the peak contains the monomer and homodimer of Y3 and STAT3ND and heterodimer between them (Fig. S2*B*). When the protein concentration increased, the molecular mass of the elution peak increased (Fig. S2*B*). When the concentrations were set to 160 μM, the molecular mass of the peak was close to that of a heterotrimer of Y3 and STAT3ND (46 kDa at a ratio of 2:1, 49 kDa at a ratio of 1:2) (Fig. S2*B*). However, this peak broadened, suggesting the simultaneous presence of various oligomers.

**Figure 3:**
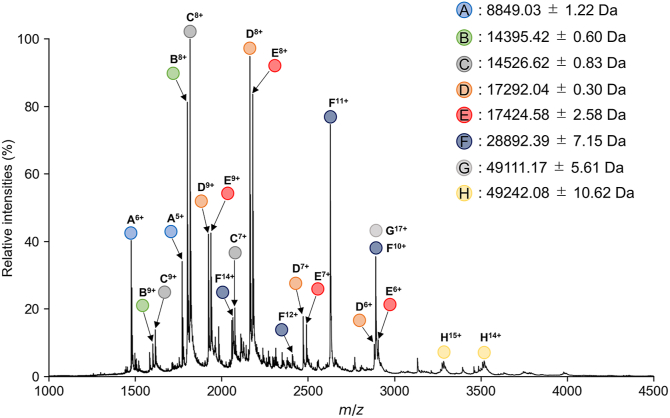
**Stoichiometric analysis of the Y3:STAT3ND complex by native MS.** Native MS spectrum of the Y3:STAT3ND complex (50 μM). The molecular mass of Y3 is calculated to be 14,526.92 Da, while that of STAT3ND is calculated to be 17,423.61 Da. (*A*) indicates a foreign substance, (*B*) indicates a monomer of Y3 lacking one amino acid residue, (*C*) indicates a monomer of Y3, (*D*) indicates a monomer of STAT3ND lacking one amino acid residue, (*E*) indicates a monomer of STAT3ND, (*F*) indicates a dimer of Y3, and (*G* and *H*) indicate a complex between Y3 and STAT3ND at a ratio of 1:2. STAT3ND, *N*-terminal domain of signal transducer and activator of transcription 3; MS, mass spectrometry.

To determine the accurate stoichiometry of the Y3:STAT3ND complex, native mass spectrometry (MS) analysis was performed using a mixed solution of Y3 and STAT3ND (Fig. S2*A*). The result of MS spectrum obtained under denaturing condition with formic acid was summarized in Text S1. Under the native condition without a denaturing agent, the native MS spectrum detected peaks of a molecular mass of approximately 49 kDa, corresponding to the Y3:STAT3ND heterotrimer formed at a molar ratio of 1:2, together with the peaks corresponding to monomers of Y3 and STAT3ND and the dimer of Y3 (Fig. 3). This result indicates that a heterotrimer can be formed by one molecule of Y3 and two molecules of STAT3ND. Unlike STAT1ND, forming a dimer seems difficult for STAT3ND (30). In fact, the unphosphorylated STAT3 is known to be prone to take a monomeric structure (31). The binding of Y3 to STAT3ND may facilitate the dimerization of STAT3 *via* stimulation of dimerization of the *N*-terminal domain. In addition, the heterotrimer formation indicates that the strong binding of the first Y3 molecule to the STAT3ND dimer induces asymmetricity in the dimer, thereby inhibiting the binding of the second Y3 molecule. These results were also confirmed by native MS experiments using Y3 and the mutant of STAT3ND taking only a monomeric form (the details were summarized in Text S2).

### Comparison of the affinities of C protein to STAT1 and STAT3

**Figure 4:**
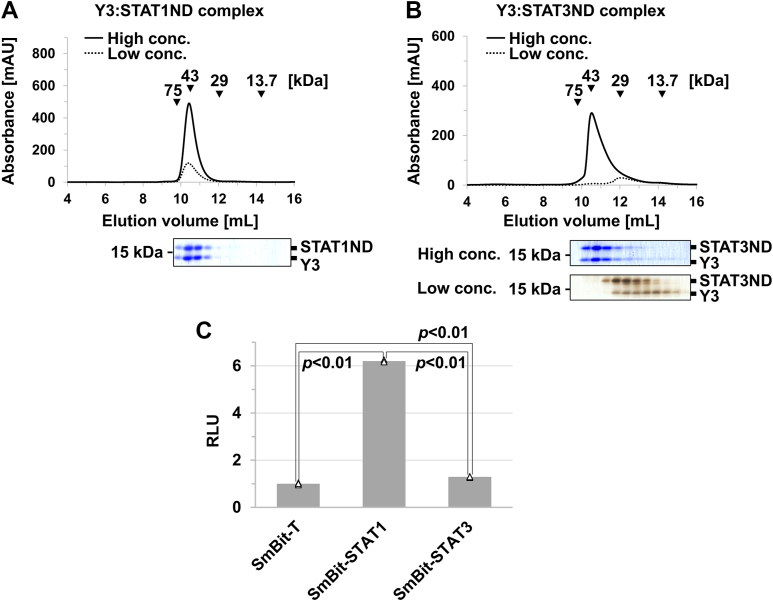
**Comparison of binding affinity between C protein and targets.***A*, SEC analysis of the Y3:STAT1ND complex at a high (130 μM) or low (10 μM) concentration. Proteins in the fractions eluted from the size-exclusion chromatograms were separated by SDS-PAGE, followed by Coomassie blue staining. *B*, SEC analysis of the Y3:STAT3ND complex at a high (130 μM) or low (10 μM) concentration. Proteins in the fractions eluted from the size-exclusion chromatograms were separated by SDS-PAGE, followed by Coomassie *blue* staining or *silver* staining. *C*, NanoBiT assay for the binding of C protein to STAT1 or STAT3. 293T cells were transfected with the expression vector for LgBiT-fused C protein, together with the expression vector for SmBiT-fused STAT1 or SmBiT-fused STAT3. The cells were also transfected with the expression vector for LgBiT-fused C protein, together with the expression vector for SmBiT-fused SV40 large T antigen as a negative control. After incubation for 24 h, cell lysates were prepared, followed by the measurement of luciferase activities. The cell lysates were also subjected to SDS-PAGE and Western blotting with an anti-GAPDH antibody. The signal intensities of GAPDH were used as internal standards. The value in the cells expressing the LgBiT-fused C protein and SmBiT-fused large T antigen was set to 1. All measured values in the bar graph are represented by *white triangles*. Error bars indicate the SD, calculated from the data of at least three experiments. *p* values were calculated on the basis of Student’s test. RLU, relative luminescence unit; STAT, signal transducer and activator of transcription; STATND, *N*-terminal domain of STAT; SmBiT, small BiT; LgBiT, large BiT; SEC, size-exclusion chromatography.

The Y3:STAT1ND complex, known to be formed at a ratio of 2:2 (12), maintained the oligomer state even under the diluted condition where the concentrations of both proteins were set at 10 μM (Fig. 4*A*). In contrast, the Y3:STAT3ND complex, which likely formed at a ratio of 1:2, was not maintained after dilution (Fig. 4*B*). Y3 may transiently and weakly bind to the STAT3ND dimer, and the release of Y3 may stimulate the dissociation of the STAT3ND dimer. In fact, the interaction between C and STAT3 could not be detected at all by the coimmunoprecipitation using the cells expressing them, unlike that between C and STAT1 (Fig. S3).

To investigate whether the full-length C protein binds to STAT3, which is expressed in culture cells, a Nanoluc Binary Technology (NanoBiT) assay was performed (32, 33). 293T cells were selected for use in the transfection experiments because several STAT3-mediated signals have been analyzed in 293T cells (34, 35, 36, 37). In this case, C protein was *C* terminally fused with a large BiT (LgBiT), whereas STAT1 or STAT3 was *N* terminally fused with a small BiT (SmBiT). When the LgBiT-fused C protein binds to the SmBiT-fused protein, Nanoluc luciferase is reconstructed between LgBiT and SmBiT, resulting in chemiluminescence in the presence of a specific substrate, furimazine. The LgBiT-fused C protein was coexpressed with SmBiT-fused STAT3 in 293T cells, followed by the preparation of the cell lysate for use in the NanoBiT assay. The luciferase activity was approximately 1.3 ± 0.1 times higher than that from cells coexpressed with SmBiT-fused SV40 large T antigen as a negative control of SmBiT fusion protein, while it was approximately 4.8 ± 0.1 times lower than that from the cells coexpressed with the SmBiT-fused STAT1 (Fig. 4*C*). These results indicate that C protein is likely to bind to STAT3 inside the cells.

### Stimulated phosphorylation of STAT3 by C protein in response to IFN-γ

IFN-γ is known to predominantly activate STAT1, whereas it only weakly activates STAT3 (38). Unlike STAT1, the activation and inactivation of STAT3 are not completely understood. If the activation process of STAT3 is the same as that of STAT1, the following scenario may occur. Upon the stimulation of IFN-γ, STAT3, which forms homodimer in a parallel fashion *via* the interaction between the *N*-terminal domains, is phosphorylated at the Tyr705 residue. Phosphorylation induces a conformational change to the scissor-like homodimer *via* interaction between the *C*-terminal regions, whereas the interaction between the *N*-terminal domains is lost (17). The phosphorylated STAT3 homodimer then enters the nucleus to promote the transcription of target genes.

To investigate the effect of the interaction between C protein and STAT3 on IFN-γ signaling *via* the STAT3 pathway, the amount of phosphorylated STAT3 in the 293T cells stimulated by IFN-γ was estimated by Western blot analysis. In this case, an expression vector of *N* terminally FLAG-tagged STAT3 (FL-STAT3) was introduced into 293T cells to increase STAT3 expression, and the amount of phosphorylated STAT3 was estimated. In the IFN-γ–stimulated cells, the amount of endogenous phosphorylated STAT3 was almost negligible compared with that of phosphorylated FL-STAT3 (Fig. S4). Therefore, the density of the band obtained by Western blot analysis using the anti-pY-STAT3 antibody appears to be related to the amount of phosphorylated FL-STAT3.

**Figure 5:**
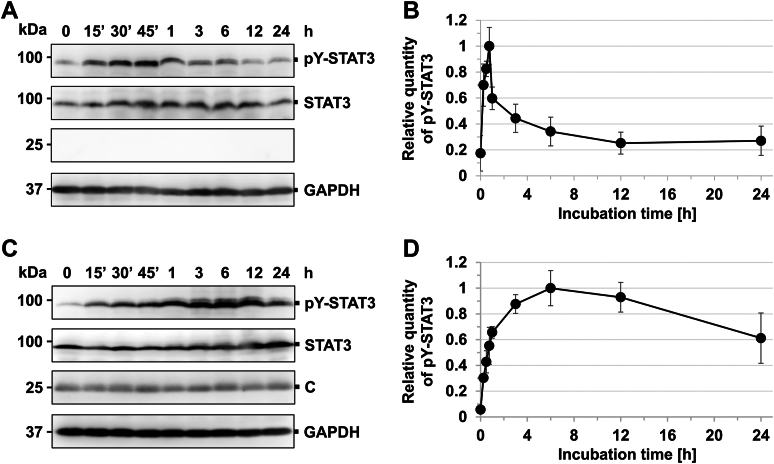
**Time course of IFN-γ–induced tyrosine phosphorylation of STAT3 in the presence of C protein.** 293T cells were transfected with the expression vector for FL-STAT3, together with an empty vector (*A*) or the expression vector for C protein (*C*). At 15, 30, or 45 min or 1, 3, 6, 12, or 24 h after the addition of IFN-γ (1000 U ml^−1^), proteins in the cell extracts were separated by SDS-PAGE and Western blot analysis with anti-STAT3, anti-phosphorylated (pY)-STAT3, and anti-GAPDH antibodies and anti-C antiserum. The relative amount of phosphorylated STAT3 was estimated on the basis of averaged signal intensities of the phosphorylated STAT3 in the absence (*B*) or presence (*D*) of C protein, calculated from three independent experiments. Band intensities were measured using ImageJ, and STAT3 signal intensities were used as internal standards. For the cells not expressing C protein, the value at 45 min after the addition of IFN-γ was set to 1. For the cells expressing C protein, the value at 6 h after the addition of IFN-γ was set to 1. Error bars indicate the SD. STAT3, signal transducer and activator of transcription 3; IFN, interferon; FL-STAT3, FLAG-tagged STAT3.

**Figure 6:**
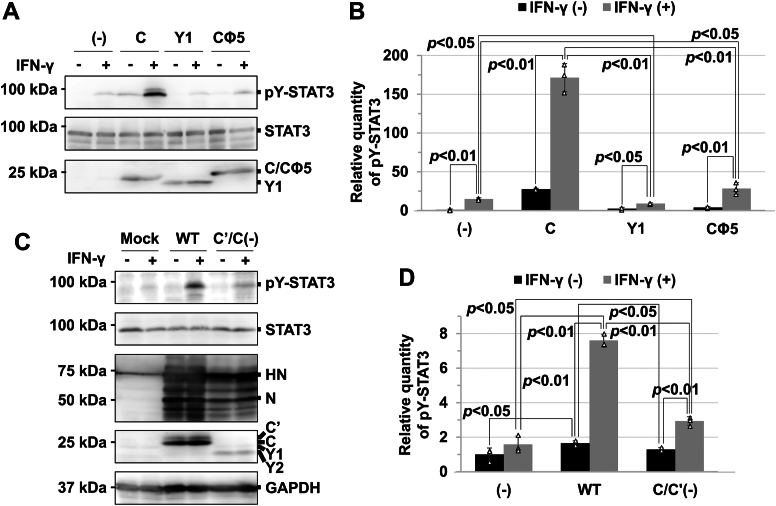
**Phosphorylation rate of STAT3 in the presence of C variants.***A*, 293T cells were transfected with the expression vector for FL-STAT3, together with an empty vector or the expression vector for C, Y1, or Cϕ5. At 3 h after the addition of IFN-γ (1000 U ml^-1^), proteins in the cell extracts were prepared, followed by SDS-PAGE and Western blot analysis with anti-STAT3 and anti-pY-STAT3 antibodies and anti-C antiserum. *B*, the relative amount of phosphorylated STAT3 was estimated on the basis of averaged signal intensities of phosphorylated STAT3 in the absence or presence of C protein variants, calculated from three independent experiments. The signal intensities of STAT3 were used as internal standards. The value in the IFN-γ–untreated cells, which were transfected with the expression vector for FL-STAT3, together with an empty vector, was set to 1. *C*, 293T cells were infected with WT SeV or a recombinant SeV, C′/C(−). After adsorption for 1 h, the cells were incubated with serum-free DMEM at 37 °C for 23 h, followed by the addition of IFN-γ (1000 U ml^-1^) to the culture medium. After additional incubation for 3 h, proteins in the cell extracts were separated by SDS-PAGE for Western blot analysis using anti-STAT3, anti-pY-STAT3, and anti-GAPDH antibodies and anti-SeV and anti-C protein antisera. After infection, the cells were also incubated at 37 °C for 27 h without the addition of IFN-γ, followed by the preparation of the cell extract for SDS-PAGE and Western blotting. *D*, the relative amount of phosphorylated STAT3 was estimated on the basis of the averaged signal intensities of phosphorylated STAT3, calculated from three independent experiments. STAT3 signal intensities were used as internal standards. The value in the IFN-γ–untreated and mock-infected cells was set to 1. All measured values in the bar graph are represented by *white triangles*. Error bars indicate the SD. *p* values were calculated on the basis of Student’s test. STAT3, signal transducer and activator of transcription 3; IFN, interferon; DMEM, Dulbecco's modified Eagle's medium; FL-STAT3, FLAG-tagged STAT3; SeV, Sendai virus.

The cell lysate was prepared at a given incubation time after the IFN-γ stimulation. The analysis showed that phosphorylated STAT3 increased immediately after IFN-γ stimulation, peaking at 45 min (Fig. 5, *A* and *B*). At 12 h poststimulation, the amount of phosphorylated STAT3 returned to basal levels. In contrast, in cells expressing C protein, the amount of phosphorylated STAT3 continued to increase and peaked at approximately 6 h poststimulation (Fig. 5, *C* and *D*). At 3 h after IFN-γ stimulation, the amount of the phosphorylated STAT3 in the cells expressing C protein was 11 times higher than that in the cells not expressing C protein (Fig. 6, *A* and *B*). Further, STAT3 in cells expressing C protein was partially phosphorylated even at the basal state (Fig. 6, *A* and *B*), suggesting that STAT3 is easily phosphorylated in the presence of C protein.

To investigate whether IFN-γ–induced phosphorylation of STAT3 is stimulated even by the virus infection, 293T cells were infected with SeV at an input multiplicity of infection of 10. At 24 h postinfection, IFN-γ was added to the medium. Western blot analysis using the cell lysate prepared after additional incubation for 3 h showed that STAT3 in SeV-infected cells was considerably phosphorylated, approximately 4.8 times higher than that in mock-infected cells (Fig. 6, *C* and *D*). In addition, STAT3 in SeV-infected cells was partially phosphorylated, even at the basal state, similar to that in cells transiently expressing C protein (Fig. 6). Meanwhile, the analysis hardly detected a significant difference between mock-infected cells with IFN-γ stimulation and those without IFN-γ stimulation (Fig. 6, *C* and *D*). These results indicate that STAT3 phosphorylation is easily stimulated by SeV infection of the cells in response to IFN-γ.

### Stimulated accumulation of STAT3 in the nucleus by C protein

**Figure 7:**
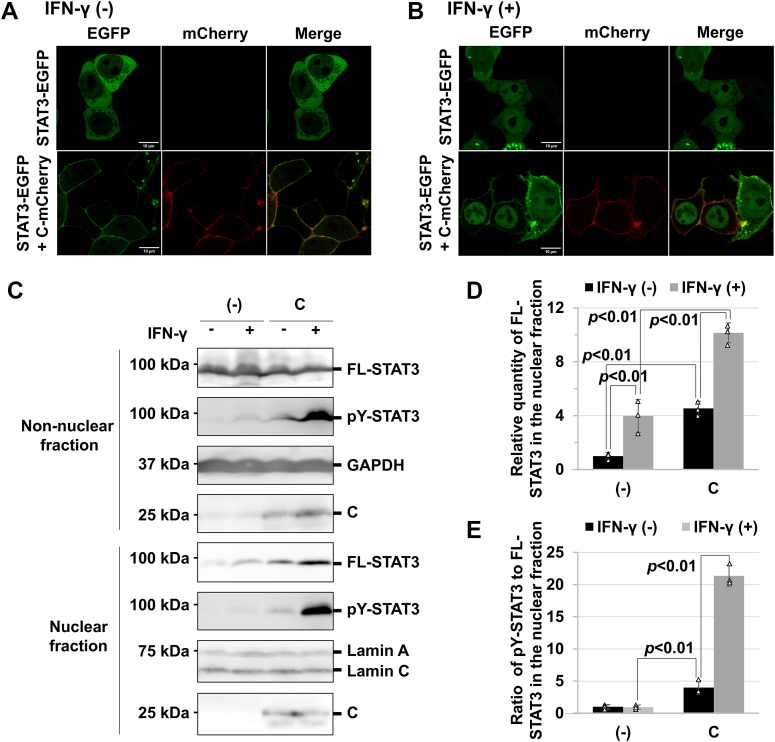
**Change of the subcellular localization of STAT3 upon stimulation of IFN-γ in the presence of C protein.***A*, 293T cells were transfected with the expression vector for STAT3-EGFP, together with an empty vector or the expression vector for C-mCherry, and protein localization was analyzed in the absence of IFN-γ. *B*, protein localization was also analyzed at 3 h after stimulation with IFN-γ (1000 U ml^−1^). The scale bar represents 10 μm. *C*, stimulation of IFN-γ–induced nuclear transition of STAT3 in the presence of C protein. 293T cells were transfected with the expression vector for FL-STAT3, together with an empty vector or the expression vector for C protein. The cells were stimulated with IFN-γ (1000 U ml^-1^) at 18 h posttransfection. After additional incubation for 6 h, nuclear and non-nuclear cell fractions were prepared, followed by SDS-PAGE and Western blot analysis with anti-FLAG, anti-pY-STAT3, anti-GAPDH, and anti-lamin A/C antibodies and anti-C antiserum. *D*, the relative nuclear transition of FL-STAT3 was estimated on the basis of averaged signal intensities of FL-STAT3 in the nucleus, calculated from three independent experiments. The signal intensities of FL-STAT3 in the non-nuclear fraction were used as internal standards. The value in IFN-γ–untreated cells without C protein was set to 1. *E*, the relative amount of phosphorylated STAT3 to FL-STAT3 in the nucleus was estimated on the basis of averaged signal intensities of pY-STAT3 in the nucleus, calculated from three independent experiments. STAT3 signal intensities in the nucleus were used as internal standards. The value in the IFN-γ–untreated cells without C protein was set to 1. All measured values in the bar graph are represented by *white triangles*. Error bars indicate the SD. *p* values were calculated on the basis of Student’s test. STAT3, signal transducer and activator of transcription 3; IFN, interferon; C-mCherry, mCherry-fused C protein; FL-STAT3, FLAG-tagged STAT3; EGFP, enhanced green fluorescent protein.

To analyze the cellular localization of C protein using confocal microscopy, the protein was *C* terminally fused with mCherry, a red fluorescent protein. The full-length C protein localizes to the plasma membrane *via* a membrane-targeting sequence (aa 1–23) at the *N* terminus (39, 40). Confocal microscopy confirmed that the mCherry-fused C protein (C-mCherry) was also localized to the plasma membrane in 293T cells (Fig. S5). Similarly, to analyze the cellular localization of STAT3 using confocal microscopy, the protein was *C* terminally fused with enhanced green fluorescent protein (EGFP). The cellular localization of EGFP-fused STAT3 (STAT3-EGFP) is almost identical to that of unfused STAT3 (41). In cells transiently expressing STAT3-EGFP, the fused protein was found to be localized predominantly to the nucleus at 3 h after IFN-γ stimulation, although it was observed in the cytosol in the absence of IFN-γ stimulation (Fig. 7, *A* and *B*).

Interestingly, in cells transiently expressing both STAT3-EGFP and C-mCherry, STAT3-EGFP was predominantly localized to the plasma membrane (Fig. 7*A*). STAT3-EGFP and C-mCherry seem to colocalize *via* interaction between STAT3ND and the Y3 region of C. The change in the cellular localization of STAT3-EGFP implies a potential role for C protein in regulating the cellular distribution of STAT3. However, at 3 h after the addition of IFN-γ to the culture medium, STAT3-EGFP was mainly found in the nucleus (Fig. 7*B*).

To estimate the amount of localized STAT3, nuclear and non-nuclear fractions were prepared from the cells transiently expressing FL-STAT3 and C at 6 h after the addition of IFN-γ. When FL-STAT3 alone was expressed, Western blot analysis showed that the amount of the nuclear FL-STAT3 in the IFN-γ–stimulated cells was 4.0 times higher than that in the unstimulated cells (Fig. 7, *C* and *D*). In contrast, the amount of nuclear FL-STAT3 in the IFN-γ–stimulated cells expressing C protein was only 2.2 times higher than that in the unstimulated cells (Fig. 7, *C* and *D*), probably because the phosphorylation of STAT3 at the basal level was increased by the expression of C protein. In total, the amount of the nuclear FL-STAT3 increased 10-fold by the synergistic effects of C and IFN-γ (Fig. 7, *C* and *D*). Similarly, the amounts of phosphorylated STAT3 in the non-nuclear fraction were found to be increased by IFN-γ stimulation (Fig. 7*C*). However, the amounts in the non-nuclear fraction were almost the same between the cells with IFN-γ stimulation in the absence of C protein and the cells without IFN-γ stimulation in the presence of C protein (Fig. 7, *C* and *D*).

Surprisingly, the ratio of phosphorylated STAT3 to FL-STAT3 in the nuclear fraction was different among the experimental conditions. The ratio in the IFN-γ–unstimulated cells with C protein was approximately 4.2-fold higher than that in the stimulated cells without C protein (Fig. 7, *C* and *E*). In the presence of C protein, the ratio increased approximately 5.3-fold by IFN-γ stimulation (Fig. 7, *C* and *E*). Thus, the introduction of C protein into the cells led to a 21-fold increase in the ratio of the amount of phosphorylated STAT3 to that of FL-STAT3 in the nucleus with IFN-γ stimulation. There was no significant difference between the ratios in the cells with or without IFN-γ stimulation in the absence of C protein. These results strongly suggest that nuclear phosphatase reactions, which catalyze the dephosphorylation of phosphorylated STAT3, are inhibited in the presence of C protein.

Although C protein mainly accumulated in the plasma membrane, Western blot analysis detected C protein in the nuclear fraction (Fig. 7*C*), indicating that C protein was translocated into the nucleus, together with phosphorylated STAT3. C protein translocating into the nucleus may inhibit the nuclear phosphatase reaction. However, C protein at the cellular membrane stimulates STAT3 phosphorylation in response to IFN-γ, resulting in an increase in the amount of phosphorylated STAT3 in the cytosol. These two effects may drastically increase the amount of phosphorylated STAT3 in the nucleus.

C protein may have a role in recruiting STAT3 to the plasma membrane, where the cytoplasmic region of the IFN-γ receptor is present, resulting in the stimulation of the IFN-γ–induced phosphorylation. Y1, which is distributed in the cytosol and nucleus owing to the lack of a membrane-targeting sequence (42), may be unable to recruit STAT3 to the plasma membrane. To investigate the effect of the membrane-targeting sequence on STAT3 phosphorylation, 293T cells expressing Y1 protein were stimulated by IFN-γ, followed by preparation of the cell lysate at 3 h poststimulation. Western blot analysis showed that the amount of phosphorylated STAT3 in the cells was significantly lower than that in the presence of C protein (Fig. 6, *A* and *B*). Similar to Y1, a mutant of C protein (Cϕ5), in which five hydrophobic residues contained in the membrane-targeting sequence are all replaced with Ala, is hardly associated with the membrane (40). In 293T cells expressing Cϕ5, although the amount of phosphorylated STAT3 after the stimulation of IFN-γ was approximately 1.9-fold higher than that in the cells not expressing C protein, it was approximately 6.0 times lower than that in the cells expressing C (Fig. 6, *A* and *B*).

To investigate the effect of the membrane-targeting sequence in SeV-infected cells, 293T cells were infected with a recombinant SeV C′/C(−) in which C′ and C expressions are knocked out (6), followed by preparation of the cell lysate after the stimulation of IFN-γ. Western blot analysis showed that the amount of phosphorylated STAT3 in the cells infected with SeV C′/C(−) was 2.6 times lower than that in the cells infected with WT SeV (Fig. 6, *C* and *D*). In addition, STAT3 at the basal level was phosphorylated less than that in cells infected with WT SeV, similar to that in mock-infected cells (Fig. 6, *C* and *D*). These results strongly indicate the importance of C protein to recruit STAT3 to the plasma membrane to stimulate the IFN-γ–induced tyrosine phosphorylation. Meanwhile, the amount of phosphorylated STAT3 increased to 2.3 times at 3 h after stimulation of IFN-γ (Fig. 6, *C* and *D*). This increase implies that STAT3 in SeV-infected cells is also phosphorylated independently of the membrane-targeting sequence of C protein.

**Figure 8:**
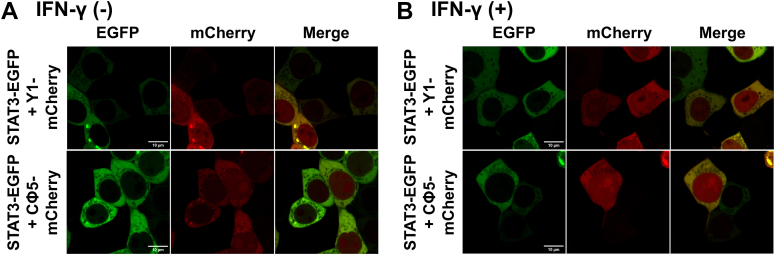
**Inhibition of IFN-γ–induced nuclear transition of STAT3 in the presence of C variants not recruiting to the plasma membrane.***A*, 293T cells were transfected with the expression vector for STAT3-EGFP, together with the expression vector for Y1-mCherry or Cϕ5-mCherry, and the protein localization was analyzed using confocal microscopy. *B*, protein localization was also analyzed at 3 h after the addition of IFN-γ (1000 U ml^−1^). The scale bar represents 10 μm. STAT3, signal transducer and activator of transcription 3; IFN, interferon; EGFP, enhanced green fluorescent protein.

Confocal microscopy analysis confirmed that Y1-mCherry and Cϕ5-mCherry were distributed in the cytosol and nucleus of 293T cells (Fig. S5). Interestingly, when STAT3-EGFP was coexpressed with Y1-mCherry or Cϕ5-mCherry, STAT3-EGFP was distributed predominantly in the cytosol but not in the membrane or nucleus (Fig. 8*A*). Even at 3 h after IFN-γ stimulation, the transition of STAT3 to the nucleus hardly increased (Fig. 8*B*). Y1 and Cϕ5 could not change the subcellular location of STAT3 from the cytosol to the nucleus, which may be related to the low efficiency of STAT3 phosphorylation in cells expressing Y1 or Cϕ5 (Figs. 6, *A* and *B*, and 8).

### Stimulation of IFN-γ–induced STAT3 signaling by C protein

**Figure 9:**
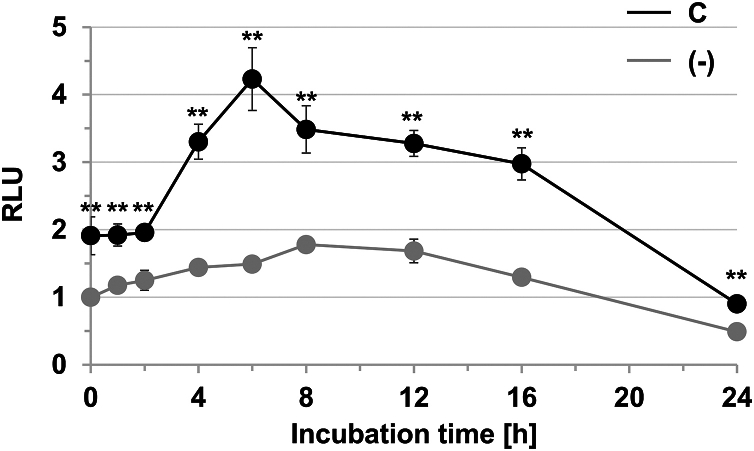
**Stimulation of IFN-γ signaling *via* the STAT3 pathway by C protein.** To estimate the strength of the response to IFN-γ, 293T cells were transfected with pSTAT3-Luc and pRL-SV40, together with an empty vector or the expression vector for C. IFN-γ (5000 U ml^−1^) was added into the culture medium at 12 h posttransfection. After additional incubation for 1, 2, 4, 6, 8, 12, 16, or 24 h, firefly luciferase activity in the cell lysates was measured. Subsequently, *Renilla* luciferase activity was measured as the internal standard. The value in the IFN-γ–untreated cells without C protein was set to 1. Error bars indicate the SD. ∗∗ indicates *p* < 0.01 when compared with the cells in the absence of C protein. *p* values were calculated on the basis of Student’s test. RLU, relative luminescence unit; IFN, interferon; STAT3, signal transducer and activator of transcription 3.

To investigate whether STAT3 signaling was stimulated by C protein, a reporter assay was conducted. 293T cells were transfected with a reporter plasmid containing the firefly luciferase gene under the control of the γ-activated sequence promoter element, which is a STAT3-binding site, together with the expression vector of C protein. The cells were collected at a given time, and luciferase activity in the cell lysate was measured. The activity at the basal level was approximately 1.9-fold higher than that in cells in the absence of C protein, which is consistent with the result that the amount of phosphorylated STAT3 at the basal level increased in the presence of C protein (Figs. 6 and 9). When C protein is expressed in the cell, STAT3 is likely to accumulate in the plasma membrane because of their mutual interaction (Fig. 7, *A* and *B*). A part of the membrane-associated STAT3 may be phosphorylated without IFN-γ stimulation, as supported by the result of the confocal analysis (Fig. S6).

In the absence of C protein, the luciferase activity in the cells peaked at 8 h after the addition of IFN-γ, which shows 1.8-fold increase from the basal level (Fig. 9). In contrast, in the cells expressing C protein, luciferase activity reached a peak at 6 h after the addition of IFN-γ, which shows a 2.2-fold increase from the basal level (Fig. 9). Although phosphorylated STAT1, which is increased by interaction with C protein, is unable to promote gene transcription (11), phosphorylated STAT3, which is increased in the presence of C protein, may promote gene transcription.

## Discussion

Upon IFN-γ stimulation, homodimers of STAT1 and STAT3 are phosphorylated, resulting in the generation of scissor-like active forms capable of promoting the transcription of the target genes. The molecular mechanisms of phosphorylation and dephosphorylation of STAT1 are the most extensively studied among the same family of proteins. Before the phosphorylation of STAT1, the homodimer transiently takes a parallel form that is easily phosphorylated by a kinase localized at the membrane (15). The interaction between the *N*-terminal domains of STAT1 is considered necessary for the generation of the parallel form, although the interactions may be lost after conversion to the scissor-like active form (43). Meanwhile, when the phosphorylated STAT1 homodimer is converted into the antiparallel form *via* the parallel form, it is easily dephosphorylated by phosphatase, resulting in the suppression of IFN-γ signaling (14, 16). Since the phosphorylation and dephosphorylation go through the parallel form, dimerization of the *N*-terminal domains may have dual roles in stimulating both reactions.

In a previous study, we proposed that when one molecule of C protein binds to the interface between the *N*-terminal domains of STAT1 in the homodimer, it tends to take an antiparallel form, which is easily dephosphorylated, resulting in the suppression of the STAT1 pathway (12). In contrast, when the two molecules of C protein bind to the STAT1 homodimer, it maintains to take a parallel form, in which is tolerant to dephosphorylation (12). However, the 2:2 complex of C protein and phosphorylated STAT1 cannot assume a scissor-like active form owing to the rigid formation of the 2:2 heterotetramer, also resulting in the suppression of the STAT1 pathway (12).

Upon stimulation of IFN-α/β, STAT1 and STAT2 pre-interact and get phosphorylated, resulting in the generation of a scissor-like active STAT1:STAT2 heterodimer capable of promoting target gene transcription (44). Considering the high sequence and structural similarities, phosphorylation and dephosphorylation of STAT family proteins may occur through the same mechanism as that of the STAT1 homodimer. C protein is known to specifically interact with STAT1ND (12). We proposed that only 1 C protein binds to the interface between STAT1ND and STAT2ND, generated in the parallel form of the phosphorylated STAT1:STAT2 heterodimer, inducing a structural change to the antiparallel form (13). The structural change stimulates dephosphorylation by phosphatase, thereby suppressing IFN-α/β signaling.

In contrast, C protein stimulates the accumulation of phosphorylated STAT3, which can promote gene transcription in response to IFN-γ signaling (Figure 5, Figure 6, Figure 7, Figure 5, Figure 6, Figure 7, and S6). Unlike STAT1, unphosphorylated STAT3 is prone to adopting a monomeric structure (31), probably because of the difficulty in forming a homodimeric structure between the *N*-terminal domains (30). C protein can bind to the parallel form of STAT3, in which the *N*-terminal domains are dimerized, stabilizing the parallel form. The parallel form is prone to phosphorylation by the kinase on the membrane (41), leading to a conformational change to the scissor-like structure in which the interactions between the *N*-terminal domains are lost (43). Unlike STAT1, the conformational change from the parallel to the scissor-like form occurs easily because of the weak interaction between C protein and STAT3ND.

The scissor-like active dimer may be inactivated by dephosphorylation *via* a conformational change to the antiparallel form. If the phosphorylated active form is more stable than the parallel form, a conformational shift to the antiparallel form is difficult. The present study indicates that the binding of C protein to STAT3ND stimulates a conformational shift of the unphosphorylated monomeric form to the parallel form but not the shift of the phosphorylated active form to the parallel form. In the case of STAT3, it would be difficult for the latter conformational shift to occur because the stabilization effect of the dimerization of the *N*-terminal domains is lower than the energy required to dissociate the interactions between the *C*-terminal fragments. Moreover, we found a possible role for C protein in inhibiting STAT3 dephosphorylation. Unlike STAT1, the interaction between C and STAT3ND may inhibit the conversion of scissor-like phosphorylated STAT3 to a parallel form.

The role of IFN-γ signaling *via* the STAT3 pathway in the viral replication and pathogenesis of SeV infection remains unclear. STAT3 is known to act as an antiviral or proviral factor, depending on the virus and host cell type (45). The present study indicates that C protein has a role in stimulating IFN-γ signal transduction *via* the STAT3 pathway, while Y1 suppresses the signaling. Relative expression levels may differ among the C variants, depending on the host, organ, and tissue infected by SeV. In contrast, C and Y1 have a common role of suppressing IFN-γ signal transduction *via* the STAT1 pathway (11), implying that suppression of the STAT1 pathway by C proteins is independent of the host and cell type.

By activating STAT1, murine native macrophages (M0) are polarized into a proinflammatory M1 type, which enhances their phagocytic capacity and ability to produce nitric oxide (46). In contrast, by activating STAT3, they are polarized into an anti-inflammatory M2 type, which promotes angiogenesis and tissue remodeling (47). C protein inhibits the signal transduction of IFN-γ *via* the STAT1 pathway, while it stimulates the signaling *via* the STAT3 pathway. Thus, C protein might also regulate the polarity of macrophages *via* the stimulation of IFN-γ produced by the surrounding immune cells. In fact, C protein is known to inhibit the functions of macrophages, including phagocytosis and the production of nitric oxide (26, 27, 28). Detailed viral infection experiments using a recombinant SeV expressing only C but not Y1 or a recombinant SeV expressing only Y1 but not C would shed light on the actual role of C proteins in the viral life cycle.

## Experimental procedures

### Cells, viruses, and antibodies

293T cells (human renal epithelial cells expressing the SV40 large T antigen; RIKEN BRC Cell Bank) were propagated in Dulbecco's modified Eagle's medium supplemented with 10% fetal bovine serum (Biological Industries) and 100 U ml^−1^ penicillin–100 μg ml^−1^ streptomycin (Thermo Fisher Scientific). The cells were routinely tested for *mycoplasma* contamination.

WT SeV derived from the cDNA of the Z strain and recombinant SeV C′/C(−), kindly provided by A. Kato, were propagated in embryonated chicken eggs. SeV titers were measured using an immunofluorescence infectious focus assay and expressed as cell-infecting units ml^−1^ (48).

Mouse mAbs against FLAG tag (M2; Sigma-Aldrich), STAT3 (124H6), and GAPDH (60004-1-Ig), rabbit mAb against STAT3 with phosphorylated Tyr705 (D3A7), and rabbit polyclonal antibody against lamin A/C were used according to the manufacturer’s instructions. Anti-SeV particles' rabbit antiserum has been described previously (48). Anti-SeV C rabbit antiserum was generated using purified Y3 with the W125A mutation as an antigen, as previously described (19).

### Plasmid construction

The construction of a plasmid for the mammalian expression of the untagged C protein (pCAG-C) has been previously described (12). The amino acid residues of C protein and its derivatives are numbered with the *N*-terminal methionine residue of C protein as 1. The cDNA fragments encoding the untagged Y1, *N* terminally FLAG-tagged STAT1, and the *N* terminally FLAG-tagged STAT3 were prepared by using PCR and subcloned into the pCAGGS vector under the control of the chicken β-actin promoter to generate pCAG-Y1, pCAG-FL-STAT1, and pCAG-FL-STAT3, respectively. To construct the expression vector of untagged Cϕ5 protein, a DNA fragment encoding C protein was amplified with a forward primer, 5′-TCAAGAATTCACCATGCCTTCAGCCGCAAAGAAGGCTGCGAAGGCGAGAGGGAGGCGCCAGGAGGACGAG-3′ (underline indicating an *Eco*RI site), and a reverse primer, 5′-AGCCGCTCGAGTTACTCTTGCACTATGTG-3′ (underline indicating a *Xho*I site). The PCR product was cloned into the *Eco*RI-*Xho*I sites of the pCAGGS vector to generate pCAG-Cϕ5. For the construction of the expression vector of *C* terminally EGFP-fused STAT3, a DNA fragment encoding STAT3 was amplified with a forward primer, 5′-CTCATCATTTTGGCAAAGACCATGGCCCAATGGAATCAGCTACAG-3′, and a reverse primer, 5′-AGATCCTGAACCACTGCCCATGGGGGAGGTAGCGCACTCC-3′, while a DNA fragment encoding EGFP was amplified with a forward primer, 5′-GGCAGTGGTTCAGGATCTGTGAGCAAGGGCGAGGAGCTGTTCA-3′, and a reverse primer, 5′-GGAAAAAGATCTGCTAGCTTACTTGTACAGCTCGTCCATGC-3′. These amplified fragments were assembled with the vector fragment, which was prepared by double digestion with *Eco*RI and *Xho*I of the pCAGGS vector, using the seamless ligation cloning extract (SLiCE) method (49) to generate pCAG-STAT3-EGFP. For the construction of the expression vectors of *C* terminally mCherry-fused C protein, *C* terminally mCherry-fused Y1, and *C* terminally mCherry-fused Cϕ5 protein, a DNA fragment encoding C was amplified with a forward primer, 5′-TCATCATTTTGGCAAAGAATTCACCATGCCTTCATTCTTAAAGAAGATTC-3′, and a reverse primer, 5′-AGATCCTGAACCACTGCCCTCTTGCACTATGTGAGCTGCCA-3′, a DNA fragment encoding Y1 was amplified with a forward primer, 5′-CTCATCATTTTGGCAAAGGAATTCACCATGTTATCGGATTCCTCGATGCTGT-3′, and a reverse primer, 5′-GAAAAAGATCTGCTAGCGAATTCCTACTTGTACAGCTCGTCCATGC-3′, and a DNA fragment encoding Cϕ5 was amplified with a forward primer, 5′-CTCATCATTTTGGCAAAGGAATTCACCATGCCTTCAGCCGCAAAGAAGGCT-3′, and a reverse primer, 5′-AGATCCTGAACCACTGCCCTCTTGCACTATGTGAGCTGCCA-3′. These amplified fragments were assembled with the vector fragment and an amplified DNA fragment encoding mCherry with a forward primer, 5′-GGCAGTGGTTCAGGATCTGTGAGCAAGGGCGAGGAGGATAAC-3′, and a reverse primer, 5′-GGAAAAAGATCTGCTAGCCTACTTGTACAGCTCGTCCATGC-3′, by using the SLiCE method to generate pCAG-C-mCherry, pCAG-Y1-mCherry, and pCAG-Cϕ5-mCherry, respectively. For the construction of the expression vector of *C* terminally LgBiT-fused C protein, a DNA fragment encoding C protein was amplified with a forward primer, 5′-CTCATCATTTTGGCAAAGAATTCACCATGCCTTCATTCTTAAAGAAGATTC-3′, and a reverse primer, 5′-CTCTTGCACTATGTGAGCTGCCA-3′. This amplified fragment was assembled with the vector fragment and an amplified DNA fragment encoding LgBiT with a forward primer, 5′-CTCACATAGTGCAAGAGTCGAGCGGTGGTGGCGGGAGCGGA-3′, a reverse primer, 5′-GGAAAAAGATCTGCTAGCTTAACTGTTGATGGTTACTCGGAACA-3′, and a DNA fragment encoding LgBiT, which was synthesized by Integrated DNA Technologies as a template (Table S1), using the SLiCE method to generate pCAG-C-LgBiT protein. For the construction of the expression vectors of *N* terminally SmBiT-fused STAT1, *N* terminally SmBiT-fused STAT3, and *N* terminally SmBiT-fused SV40 large T antigen (SmBiT-T), a DNA fragment encoding STAT1 was amplified with a forward primer, 5′-GTGGAGGTGGTACCAGTTCTCAGTGGTACGAACTTCAGCA-3′, and a reverse primer, 5′-GGAAAAAGATCTGCTAGCCTATACTGTGTTCATCATACTGT-3′, a DNA fragment encoding STAT3 was amplified with a forward primer, 5′-GTGGAGGTGGTACCAGTGCCCAATGGAATCAGCTACAGCA-3′, and a reverse primer, 5′-GGAAAAAGATCTGCTAGCCTATACTGTGTTCATCATACTGT-3′, while a DNA fragment encoding SV40 large T antigen was amplified with a forward primer, 5′-GTGGAGGTGGTACCAGTGGAACTGATGAATGGGAGCAGT-3′, and a reverse primer, 5′-GGAAAAAGATCTGCTAGCTCATGTTTCAGGTTCAGGGGGAGGTGT-3′. The amplified fragments were assembled with the vector fragment and a DNA fragment encoding SmBiT, which was synthesized by Integrated DNA Technologies (Table S1), using the SLiCE method, to generate pCAG-SmBiT-STAT1, pCAG-SmBiT-STAT3, and pCAG-SmBiT-T, respectively.

Plasmid construction for the bacterial expression of *N* terminally histidine-tagged Y3 has been described previously (12, 19). For the expression of *N* terminally histidine-tagged STAT3ND, DNA fragments encoding STAT3 (aa 3–126 and 3–138) were cloned into the *Nde*I–*Xho*I sites of the modified pCold ProS2 vector (12) to generate pCold-STAT3ND (aa 3–126) and pCold-STAT3ND (aa 3–138), respectively. For the expression of STAT3ND (aa 4–138) fused with a small ubiquitin-related modifier (SUMO) tag at the *N* terminus, a DNA fragment encoding STAT3β was amplified with a forward primer, 5′-CGCACAATGGAATCAGCTACAGCAGCTTGACACACGGTACCTGGAGCAGCTCCATCAGCT-3′, and a reverse primer, 5′-GCTTGAATTCGGATCCCCATATGTTATTTCCAAACTGCATCAATGAATGGT-3′, while a DNA fragment encoding SUMO was amplified with a forward primer, 5′-TCCATCATCATCATCACGACTCCGAGGTGAATCAGGAGGCCAA-3′, and a reverse primer, 5′-CTGATTCCATTGTGCGCCCTGGAAGTAAAGATTTTCGCCTCCGATTTGTTCTCTGTGT-3’. These amplified fragments were assembled with the vector fragment, which was prepared by double-digestion of the modified pCold ProS2 vector with *Nde*I and *Xho*I, using the SLiCE method to generate pCold-SUMO-STAT3β. The resultant plasmid was then amplified with a forward primer, 5′-TGGTGACGTAGCTCGAGGGATCCGAATTCAAGCTT-3′, and a reverse primer, 5′-CTCGAGCTACGTCACCACGGCTGCTGTGGGAT-3′, followed by self-assembly using the SLiCE method to generate pCold-SUMO-STAT3ND. The L78R mutant of SUMO-STAT3ND was generated by introducing the mutation into the expression vector for SUMO-STAT3ND by using a KOD-Plus-Mutagenesis Kit (Toyobo). Mutation was confirmed by DNA sequencing analysis.

As prey for the yeast two-hybrid analysis, DNA fragments encoding STAT1, STAT2, STAT3, STAT3ND (aa 1–126), STAT3 lacking the *N*-terminal domain (STAT3ΔND; aa 127–851), TYK2, and JAK1 were cloned into the *Nde*I–*Xho*I sites of pGADT7 vector, respectively. For use as bait for the yeast two-hybrid analysis, DNA fragments encoding C and Y3 were cloned into the *Nde*I–*Bam*HI sites of the pGBKT7 vector.

### Yeast two-hybrid analysis

Yeast transformation and two-hybrid analysis were performed using the Matchmaker Gold Yeast Two-Hybrid System (Takara) according to the manufacturer’s instructions. *Saccharomyces cerevisiae* strain Y187 was transformed with pGADT7 or pGADT7 carrying the prey protein by lithium acetate transformation, whereas the Y2H Gold strain was transformed with pGBKT7 or pGBKT7 carrying the bait protein. The hybrid strains were grown at 30 °C for 4 days on SD/–Leu/–Trp agar media supplemented with or without 200 ng ml^−1^ aureobasidin A and 40 ng ml^−1^ X-α-gal, which is converted into a blue pigment by α-galactosidase secreted *via* the interaction between bait and prey.

### Protein preparations

Y3, STAT3ND, and SUMO-STAT3ND variants were expressed in *E*. *coli* BL21(DE3) (Novagen) at 15 °C for 24 h after induction with 0.2 mM IPTG. All proteins possessing the histidine tag were purified by nickel affinity chromatography using Cosmogel His-Accept (Nacalai Tesque) according to the manufacturer’s instructions. Proteins were further purified by SEC using a HiLoad Superdex 75 prep grade 16/600 column (GE Healthcare Life Sciences) and 20 mM Tris–HCl buffer (pH 8.0) containing 100 mM NaCl, 1 mM EDTA, 1 mM DTT, and 5% glycerol as a running buffer. Nickel affinity chromatography was also used to prepare Y3:STAT1ND and Y3:STAT3ND complexes after mixing the supernatant from Y3-expressed *E. coli* with that from STAT1ND-expressed *E. coli* and that from STAT3ND-expressed *E. coli*, respectively. The complexes were then separated from the uncomplexed Y3 by SEC using a HiLoad Superdex 75 prep grade 16/600 column equilibrated with the running buffer.

The protein concentrations of Y3, STAT3ND, SUMO-STAT3ND variants, and Y3:STAT1ND and Y3:STAT3ND complexes were determined by measuring the absorbance at 280 nm using molar extinction coefficients of 22,100, 30,940, 33,920, 46,400, and 53,400 M^−1^ cm^−1^, respectively.

### SEC analysis

Prior to SEC analysis, Y3 (100 μM) was preincubated with STAT3ND (100 μM) for 10 min on ice. A portion of the solution was injected into a Superdex 75 Increase 10/300 GL gel filtration column (GE Healthcare) equilibrated with 20 mM Tris–HCl buffer (pH 8.0) containing 100 mM NaCl at 4 °C at a flow rate of 0.5 ml min^−1^. Y3 and STAT3ND in each fraction were resolved using SDS-PAGE and detected by Coomassie blue or silver staining. Meanwhile, the purified SUMO-STAT3ND (15 μM) or the L78R mutant (15 μM) was mixed with Y3 (30 μM), and the mixture was preincubated at 4 °C for 10 min, followed by SEC analysis using the Superdex 200 Increase 10/300 GL gel filtration column.

SEC with MALS was performed using the DAWN HELEOS2 MALS instrument (Wyatt Technology Corporation) downstream of a Waters Alliance liquid chromatography system connected to a Superdex 200 Increase 5/150 GL (Cytiva) gel filtration column. The differential refractive index downstream of the MALS detector was used to determine the protein concentration. The column was equilibrated with 20 mM Tris–HCl buffer (pH 8.0) containing 100 mM NaCl, 1 mM EDTA, 1 mM DTT, and 5% glycerol. The flow rate was set at 0.15 ml min^-1^. Thirty microliters of the sample at concentrations of 8, 32, 95, or 160 μM was injected. Data were analyzed using ASTRA version 6 (Wyatt Technology Corporation; https://www.wyatt.com/products/software/astra.html).

### Native MS analysis

For native MS analysis, Y3 (50 μM) complexed with STAT3ND (50 μM), SUMO-STAT3ND (50 μM), or the L78R mutant (50 μM) was buffer-exchanged to 450 mM ammonium acetate (pH 8.0) with Micro Bio-Spin 6 columns (Bio-Rad). For analysis under denaturing conditions, 30% (*v/v*) formic acid was added to the samples, followed by buffer exchange. Prepared samples (5 μl) were loaded in gold-coated glass capillaries made in-house and immediately electro-sprayed into a Quadrupole-TOF mass spectrometer, SYNAPT G2-*Si* HDMS (Waters) by nano-ESI. Mass spectra were acquired in positive ion mode using the following instrument parameters described below (capillary voltage: 1.33 kV, sampling cone voltage: 150 V, source offset voltage: 150 V, trap collision energy: 0 V, transfer collision energy: 0 V, and trap gas flow: 2 ml min^−1^) and analyzed using MassLynx 4.1 software (Waters; https://help.waters.com/help/en/support/library-details.html?documentid=716001833&backtosearch=%2Fnextgen%2Fus%2Fen%2Fsearch.html%3Fcategory%3DSupport%2BLibrary%26enableHL%3Dtrue%26isocode%3Den_US%26keyword%3Dmasslynx%2Bversion%2B4.1%26multiselect%3Dtrue%26page%3D1%26rows%3D12%26sort%3Dmost-relevant). The mass spectrometer was calibrated using cesium iodide at a concentration of 2 mg ml^−1^ dissolved in 50% 2-propanol in advance of the analysis.

### Coimmunoprecipitation and Western blotting

Subconfluent 293T cells in a 12-well plate were transfected with the indicated plasmids (0.50 μg each) using FuGENE HD reagent (Promega). After 24 h, the cells were solubilized in 0.25 ml of the NP-40 lysis buffer [1% NP-40, 25 mM Tris–HCl (pH 7.6), 150 mM NaCl, 1 mM EDTA, 5% glycerol, and Complete Mini Protease Inhibitor Cocktail (Roche Diagnostics)]. Cell lysates were immunoprecipitated with an anti-FLAG antibody and protein G Sepharose (GE Healthcare). The immunoprecipitates were washed three times with cell lysis buffer and once with wash II buffer [50 mM Tris–HCl (pH 7.4), 50 mM NaCl, and 5 mM EDTA] and then analyzed by Western blotting using an anti-FLAG antibody or an anti-C antiserum after separation by SDS-PAGE. Protein bands were detected by using horseradish peroxidase (HRP)-conjugated mouse IgGκ light chain binding protein (sc-516102; Santa Cruz Biotechnology) or HRP-conjugated anti-rabbit IgG mouse mAb (sc-2357; Santa Cruz Biotechnology) as a secondary antibody and Luminata Forte Western HRP Substrate (Millipore), followed by analysis using LAS-4000 Lumino image analyzer (Fujifilm). A portion of the cell lysate was also processed for SDS-PAGE and Western blotting to confirm protein expression.

### Nuclear localization analysis

To estimate the nuclear transition rate of STAT3, 30% confluent 293T cells in a 6-well plate were transfected with the indicated plasmids (1.0 μg each) using FuGENE HD reagent. At 18 h posttransfection, IFN-γ (1000 U ml^−1^) was added into the culture medium. After additional incubation for 6 h, cytosolic and nuclear fractions were prepared using a LysoPure Nuclear and Cytoplasmic Extractor Kit (Wako) according to the manufacturer’s instructions, followed by SDS-PAGE and Western blot analysis.

### NanoBiT assay for protein–protein interactions

For quantitative analysis of the interaction between C protein and STAT1 or STAT3 by NanoBiT assay, subconfluent 293T cells in a 24-well plate were transfected with the indicated plasmids (0.25 μg each) using FuGENE HD reagent. After 24 h, the cells were solubilized in 0.20 ml of the NP-40 lysis buffer, and luciferase activity was measured using the Nano-Glo Luciferase Assay System (Promega) with an EnSight multimode plate reader according to the suppliers’ instructions. Cell lysates were subjected to SDS-PAGE and Western blotting using an anti-GAPDH antibody. The signal intensities of GAPDH were used as the internal standards. SDs were calculated from the data obtained from at least three experiments.

### Measurement of pY-STAT3 in SeV-infected cells

Thirty percent confluent 293T cells cultured on collagen-coated wells in a 12-well plate were infected with SeV at an input multiplicity of infection of 10. After adsorption at 37 °C for 1 h, the inoculum was removed, and 1 ml of serum-free Dulbecco's modified Eagle's medium was added. At 24 h postinfection, IFN-γ (1000 U ml^−1^) was added into the culture medium. After additional incubation for 3 h, cell lysates were prepared for SDS-PAGE and Western blotting.

### Confocal microscopy

For confocal microscopy, subconfluent 293T cells cultured on collagen-coated wells in an 8-well plate were transfected with the indicated plasmids (0.25 μg each) using FuGENE HD reagent. The cells were further incubated at 37 °C for 21 h, followed by the addition of IFN-γ (1000 U ml^-1^). After additional incubation for 3 h, subcellular localization of the indicated proteins was analyzed using a confocal microscope FV1000 (Olympus) equipped with a temperature- and CO_2_-controlled stage chamber. Images were processed using ImageJ (50) with the Fiji package (51). For the visualization of phosphorylated STAT3 in the cells, subconfluent 293T cells cultured on collagen-coated cover slips in a 12-well plate were transfected with the indicated plasmids (0.50 μg each). The cells were further incubated at 37 °C for 21 h, followed by the addition of IFN-γ (1000 U ml^−1^). After incubation for 3 h, the cells were fixed with 4% paraformaldehyde in PBS for 20 min and permeabilized with 0.2% Triton X-100 in PBS for 20 min. The samples were mounted with Fluoromount-G and 4′,6-diamidino-2-phenylindole (Southern Biotech) and placed on glass slides. The subcellular localization of STAT3-EGFP was identified based on the fluorescent signal from EGFP, while those of C-mCherry, Y1-mCherry, and Cϕ5-mCherry were identified based on the fluorescent signal from mCherry. Phosphorylated STAT3 was visualized using immunostaining. Fixed and permeabilized cells were treated with an antiphosphorylated Stat3 antibody (4 °C, overnight), followed by a secondary antibody reaction using a goat anti-rabbit IgG-Alexa Fluor 647 conjugate at room temperature for 3 h.

### Reporter assay

For IFN-γ signal transduction *via* the STAT3 pathway, 30% confluent 293T cells in a 24-well plate were transfected with pSTAT3-Luc (0.25 μg, Panomics) and pRL-SV40 (0.025 μg, Promega), together with the indicated plasmid (0.25 μg each) using the FuGENE HD reagent. The cells were further incubated at 37 °C for 12 h, followed by the addition of IFN-γ (5000 U ml^−1^). After additional incubation for the indicated times, firefly luciferase activity was measured using the Dual-Luciferase Reporter Assay System (Promega) with the EnSight multimode plate reader, according to the supplier’s instructions. *Renilla* luciferase activity was measured as an internal standard. SDs were calculated from the data obtained from at least three experiments.

## Data availability

The data described in the article are contained within the article and supplementary files.

## Supporting information

This article contains supporting information (52).

## Conflicts of interest

The authors declare that they have no conflicts of interest with the contents of this article.
