# Supplementary material for: Molecular basis of IFN-γ–induced STAT3 phosphorylation stimulated by Sendai virus C protein
Source: J Biol Chem. 2025 Sep 18;301(11):110744. doi: 10.1016/j.jbc.2025.110744 (PMC12552534; doi:10.1016/j.jbc.2025.110744)
Supplement: Supporting information [file mmc1.pdf]

Molecular basis of IFN-γ-induced STAT3 phosphorylation stimulated by Sendai virus C protein

Kosuke Oda[1,2,*], Yuta Hatori[1], Atsuji Kodama[3], Susumu Uchiyama[3,4], Takashi Oda[5,§], Yasuyuki Matoba[1], Ami Nakano[1,‡], Kanako Ninomiya[1,‡], Seira Yoshidomi[1,‡], Takemasa Sakaguchi[2]

[1]Faculty of Pharmacy, Yasuda Women's University, 6-13-1 Yasuhigashi, Asaminami-ku, Hiroshima 731-0153, Japan

[2]Department of Virology, Institute of Biomedical and Health Sciences, Hiroshima University, 1-2-3 Kasumi, Minami-ku, Hiroshima 734-8553, Japan

[3]Exploratory Research Center on Life and Living Systems (ExCELLS), National Institutes of Natural Sciences, 5-1 Higashiyama, Myodaiji, Okazaki, 444-8787, Japan

[4]Department of Biotechnology, Graduate School of Engineering, Osaka University, 2-1 Yamadaoka, Suita, Osaka, 565-0871, Japan.

[5]Department of Life Science, Rikkyo University, 3-34-1 Nishi-Ikebukuro, Toshima-ku, Tokyo, 171-8501, Japan

[§]Current address: Materials and Life Science Division, J-PARC Center, Japan Atomic

Energy Agency, 2-4 Shirakata, Tokai, Ibaraki, 319-1195, Japan.

‡These authors contribute equally to this work.

*Correspondence: oda-k@yasuda-u.ac.jp

Materials included:

Figure S1 to Figure S14

Table S1

Supplementary Text S1 to Supplementary Text S2

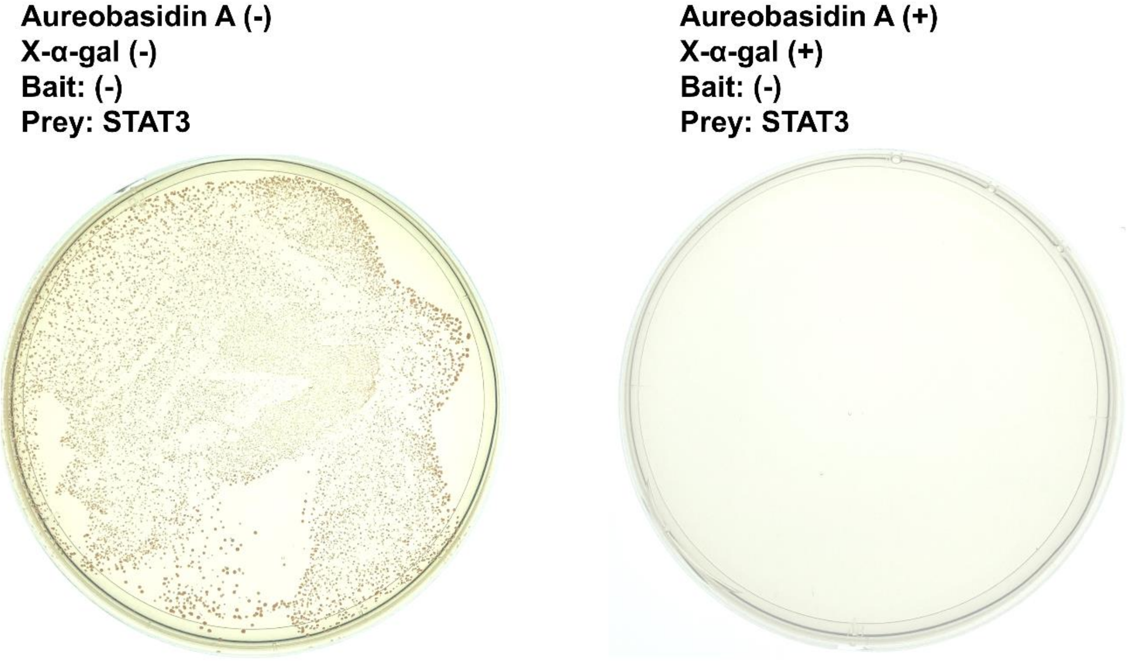

Figure S1. Confirmation of no false positive reaction in the yeast two-hybrid experiment when using STAT3 as prey. Yeast two-hybrid analysis using the GAL4 binding domain unfused to C protein as a negative bait protein and GAL4 activation domain-fused STAT3 as prey. Experiments were performed in the absence or presence of aureobasidin A and X-α-gal for colony selection.

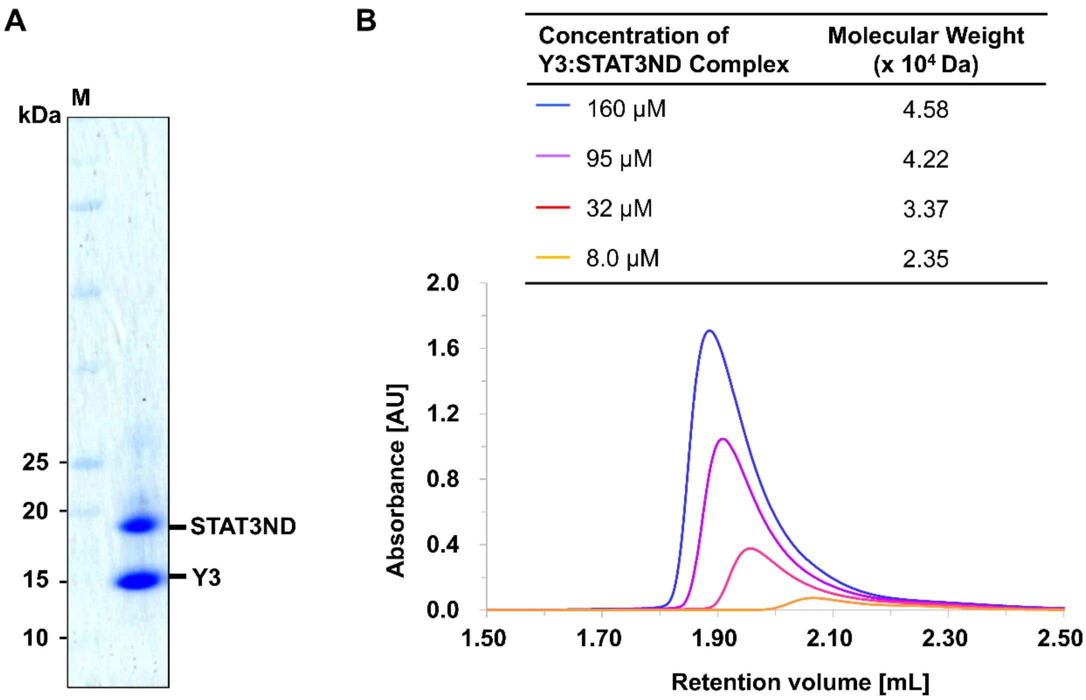

Figure S2. SEC analysis with MALS using the Y3:STAT3ND complex. *A*, SDS-PAGE analysis of Y3 and STAT3ND used for SEC analysis with MALS. *B*, Molecular mass of the elution peak estimated by SEC-MALS when the concentration of the Y3:STAT3ND complex was set to 160 µM (blue line), 95 µM (purple line), 32 µM (red line), or 8 µM (yellow line).

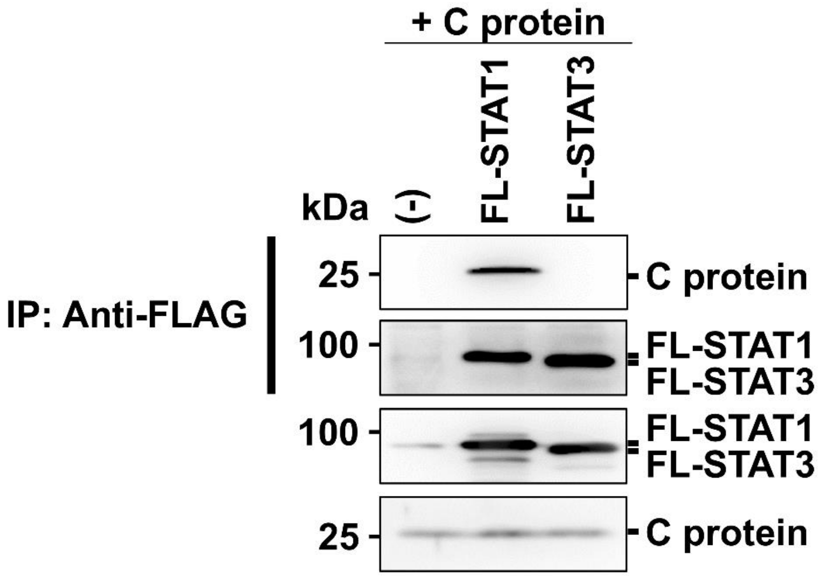

Figure S3. Co-immunoprecipitation analysis between C protein and targets. For the co-immunoprecipitation of C protein with FL-STAT1 or FL-STAT3, 293T cells were transfected with an expression vector for C protein, together with that for FL-STAT1 or FL-STAT3 or an empty vector. Proteins in the cell lysates were immunoprecipitated with an anti-FLAG antibody and analyzed by western blotting using an anti-FLAG antibody and anti-C antiserum.

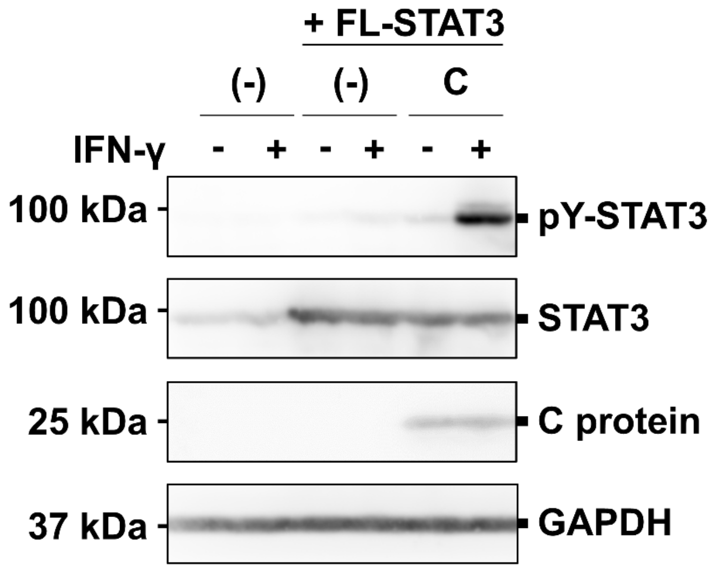

Figure S4. Stimulation of IFN-γ-induced tyrosine phosphorylation of STAT3 in the presence of C protein. 293T cells were transfected with the expression vector for FL-STAT3, together with an empty vector or the expression vector for C protein. The cells were also transfected with the empty vector alone. At 3 h after the addition of IFN-γ (1,000 U mL^{-1}), proteins in the cell extracts were prepared, followed by SDS-PAGE and western blot analysis with anti-STAT3, anti-phosphorylated (pY)-STAT3, and anti-GAPDH antibodies and anti-C antiserum.

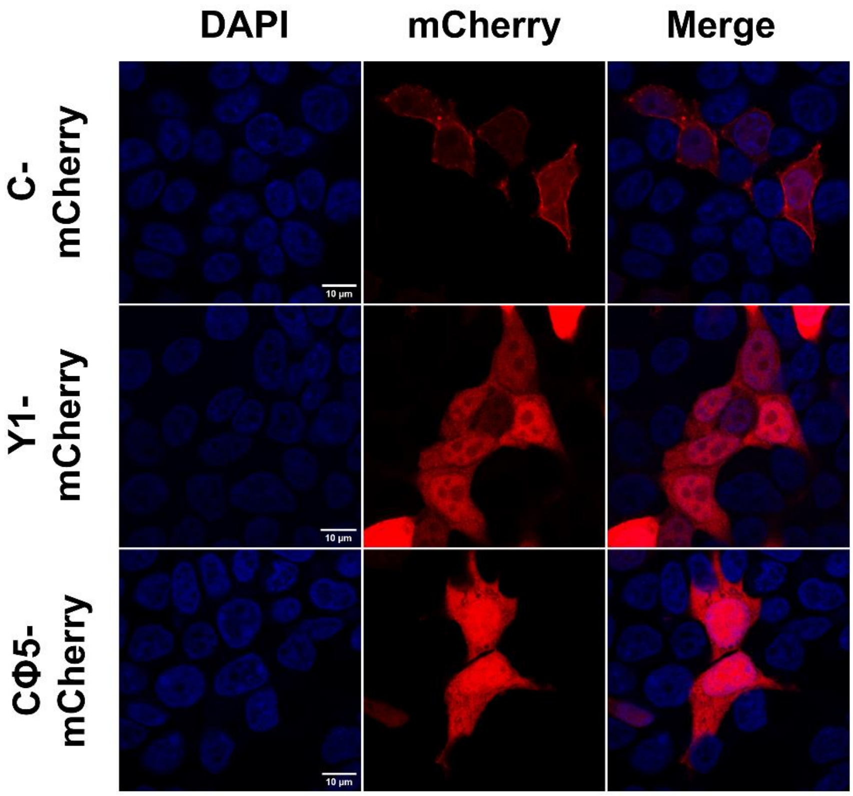

Figure S5. Subcellular localization of C variants. 293T cells were transfected with the expression vector for C-mCherry, Y1-mCherry, or Cϕ5-mCherry, and fixed with 4% paraformaldehyde, followed by permeabilization and mounting with DAPI. Protein localization was analyzed using confocal microscopy. Scale bar, 10 μm.

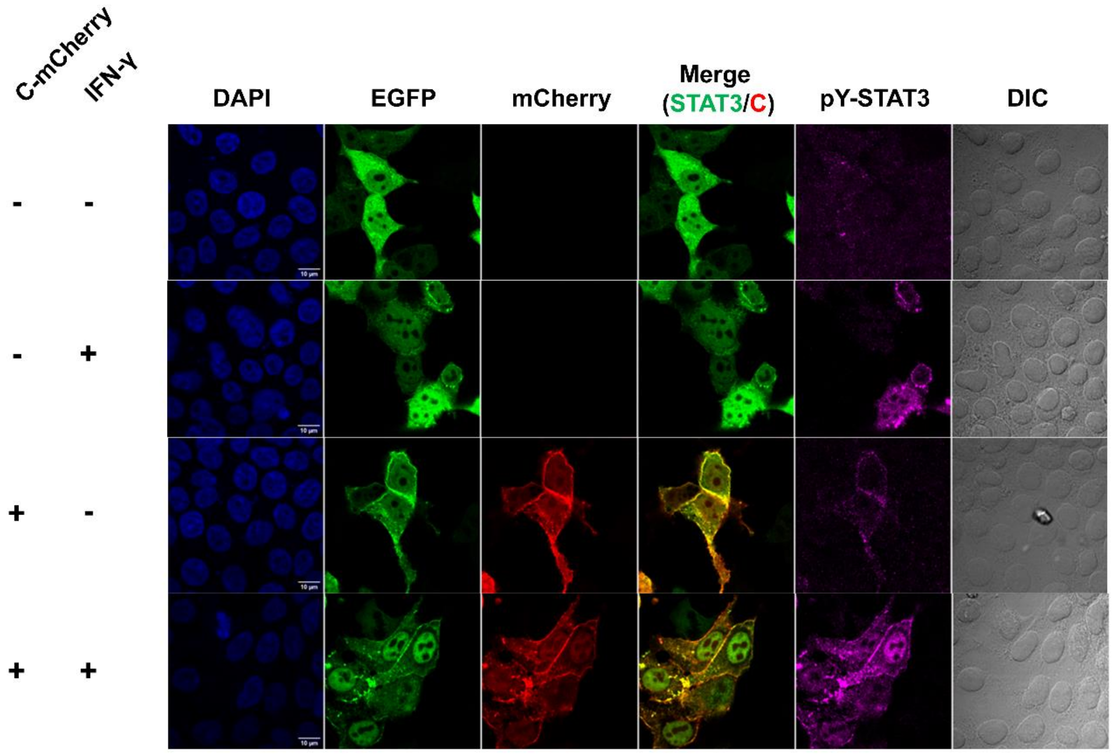

Figure S6. Subcellular localization of phosphorylated STAT3 in the presence of C protein after IFN-γ stimulation. 293T cells were transfected with the expression vector for STAT3-EGFP, together with an empty vector or the expression vector for C-mCherry, and fixed with 4% paraformaldehyde at 3 h after stimulation with IFN-γ (1,000 U mL^{-1}), followed by permeabilization, immunostaining with an anti-pY-STAT3 antibody, and confocal microscopy analysis. Protein localization was also analyzed in the absence of IFN-γ stimulation. Scale bar, 10 μm. DIC, differential interference contrast.

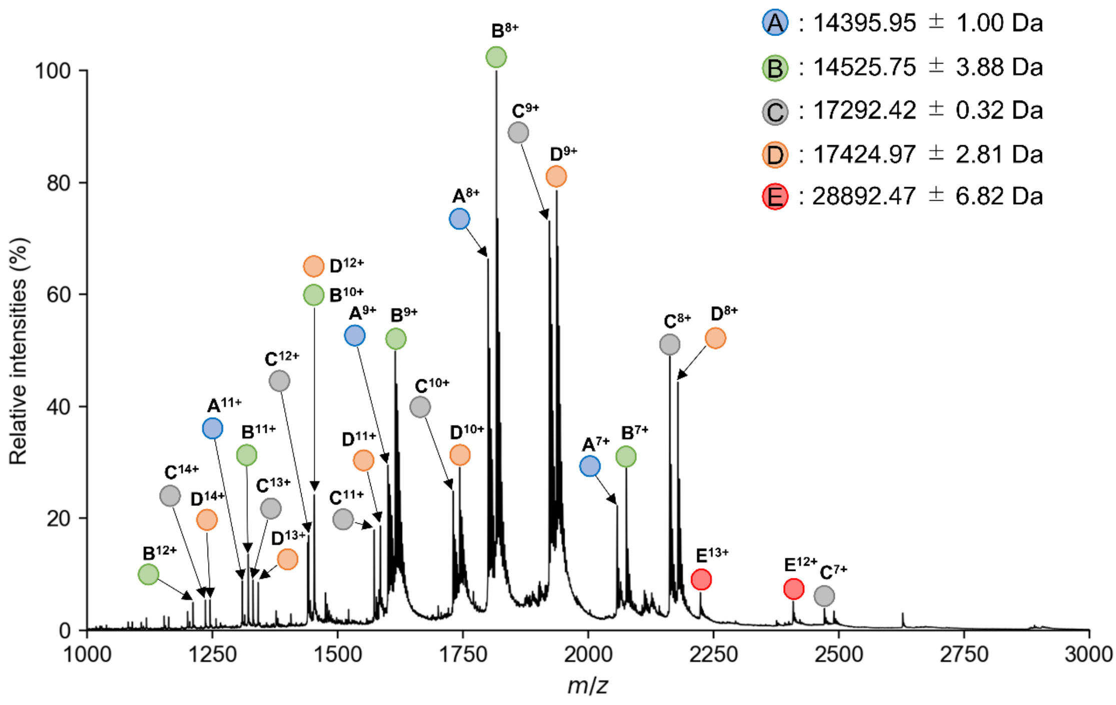

Figure S7. MS analysis of the Y3:STAT3ND complex under denaturing condition.

Mass spectrum of the Y3:STAT3ND complex (50 μM) under denaturing condition with formic acid. The molecular mass of Y3 is calculated to be 14,526.92 Da, while that of STAT3ND is calculated to be 17,423.61 Da. A indicates a monomer of Y3 lacking one amino acid residue, B indicates a monomer of Y3, C indicates a monomer of STAT3ND lacking one amino acid residue, D indicates a monomer of STAT3ND, and E indicates a dimer of Y3.

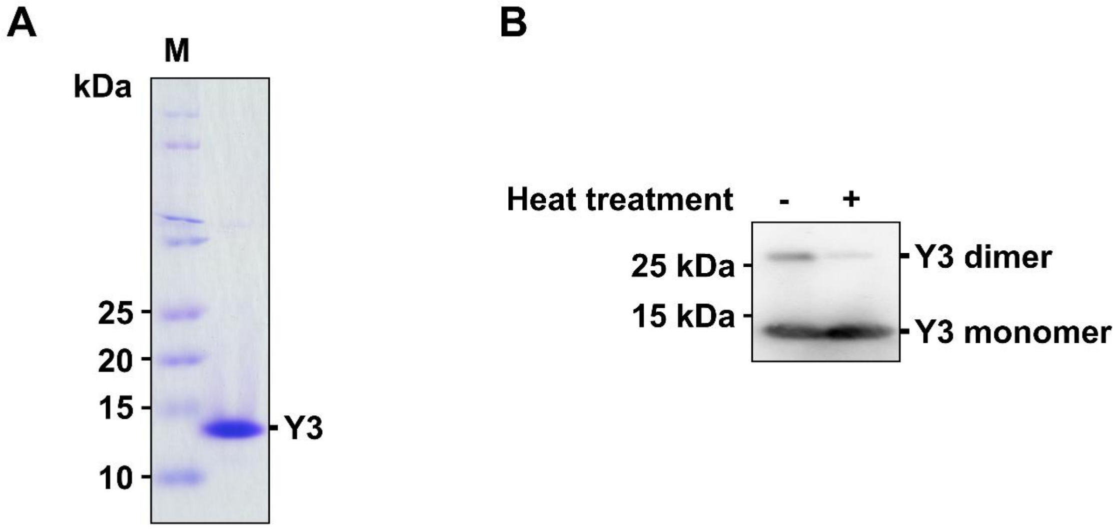

Figure S8. Partial homodimerization of Y3. *A*, SDS-PAGE analysis of Y3 used for the homodimerization analysis. *B*, Heat-treated or -untreated Y3 (60 μM) was processed by SDS-PAGE and western blotting using anti-C antiserum.

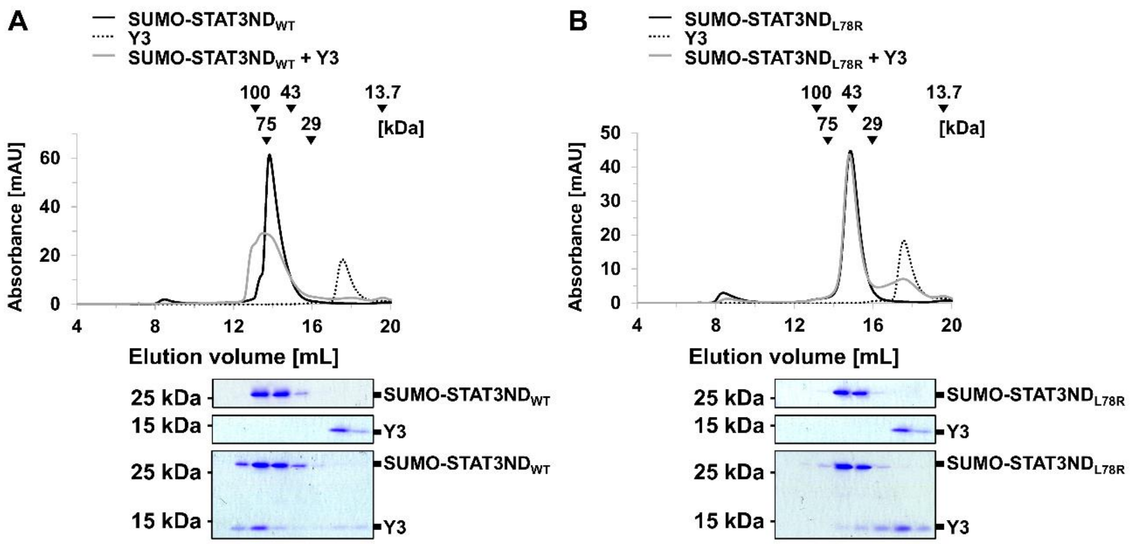

Figure S9. Binding assay between SUMO-STAT3ND and Y3. SEC analysis of SUMO-STAT3ND (*A*) or the L78R mutant (*B*) in the absence (black line) or presence (gray line) of Y3. SUMO-STAT3ND (15 μM) was pre-incubated with or without Y3 (30 μM) for 10 min, followed by SEC analysis. A chromatogram of Y3 alone is shown as a dashed line. Proteins in the fractions eluted from the size-exclusion chromatograms were separated by SDS-PAGE, followed by Coomassie blue staining.

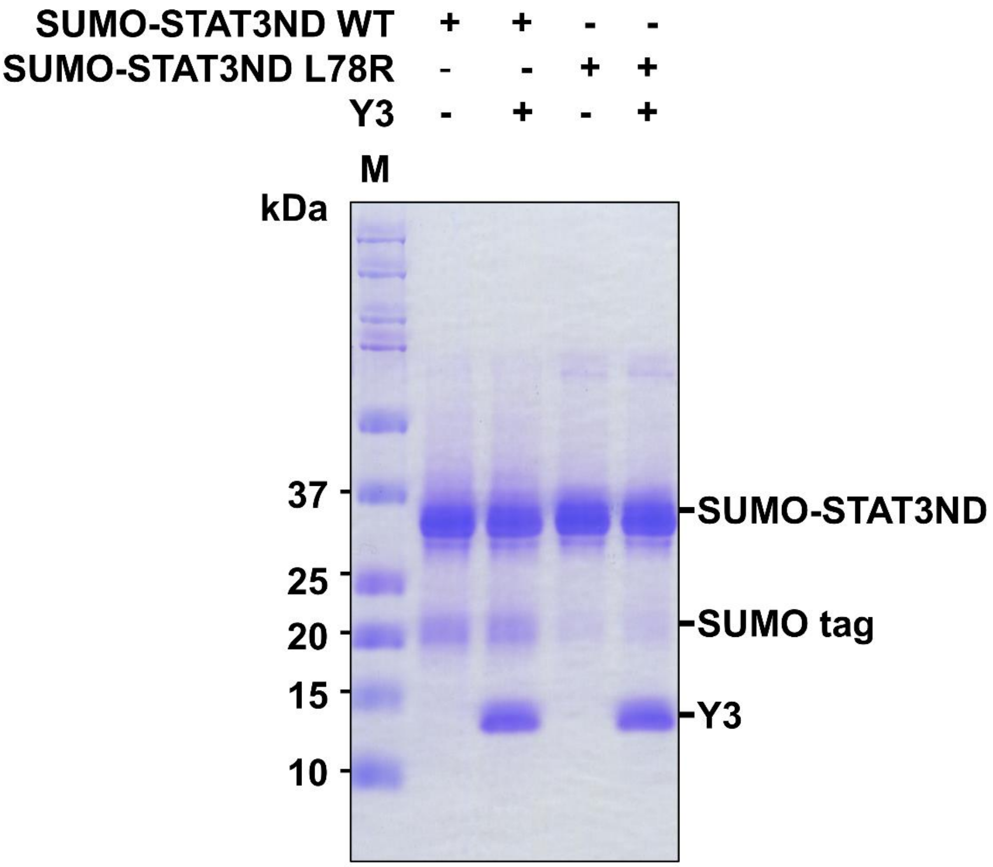

Figure S10. SDS-PAGE analysis of wild-type and L78R mutant of SUMO-STAT3ND complexed with or without Y3 used for native MS.

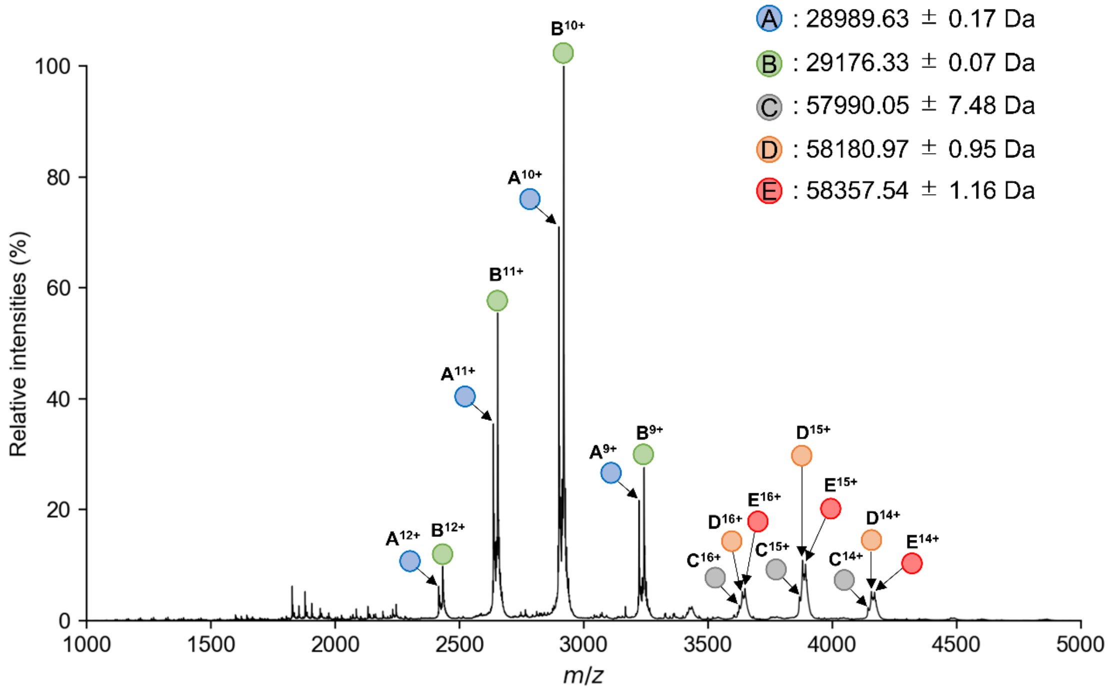

Figure S11. Stoichiometric analysis of SUMO-STAT3ND by native MS. Native MS spectrum of SUMO-STAT3ND (50 μM). The molecular mass of SUMO-STAT3ND is calculated to be 29,090.63 Da. A and B indicate a monomer of SUMO-STAT3ND. C, D, and E indicate a dimer of SUMO-STAT3ND.

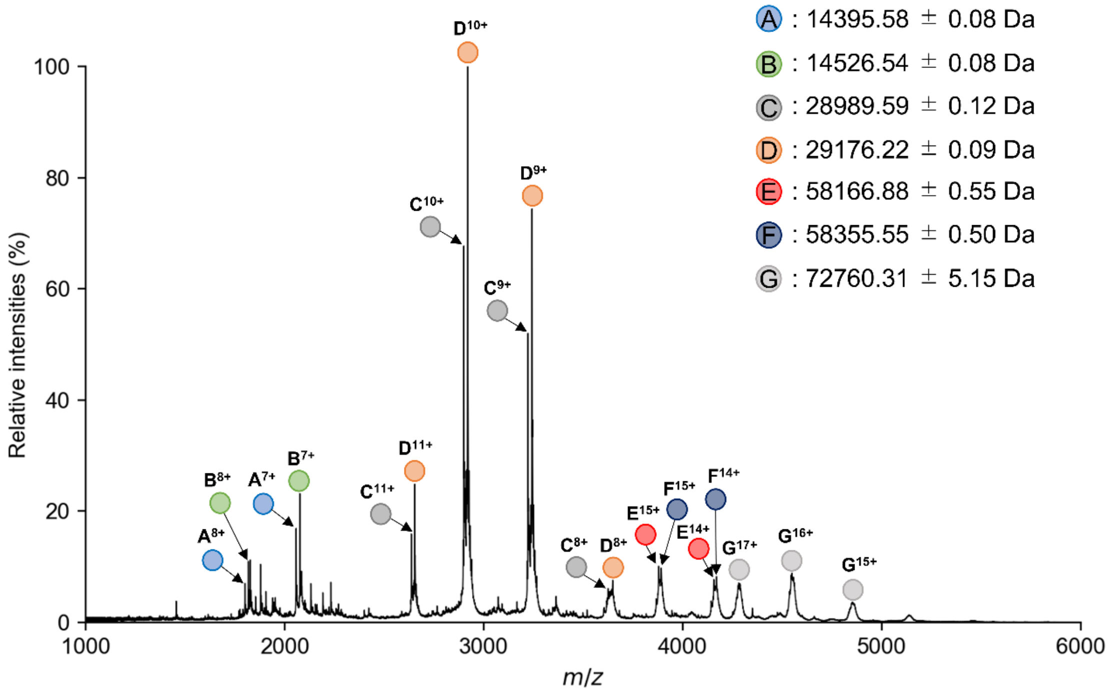

Figure S12. Stoichiometric analysis of the Y3:SUMO-STAT3ND complex by native MS. Native MS spectrum of the Y3:SUMO-STAT3ND complex (50 μM). The molecular mass of Y3 is calculated to be 14,526.92 Da, while that of SUMO-STAT3ND is calculated to be 29,090.63 Da. A indicates a monomer of Y3 lacking one amino acid residue, B indicates a monomer of Y3, C and D indicate a monomer of SUMO-STAT3ND, E and F indicate a dimer of SUMO-STAT3ND, and G indicates a complex between Y3 and SUMO-STAT3ND at a ratio of 1:2.

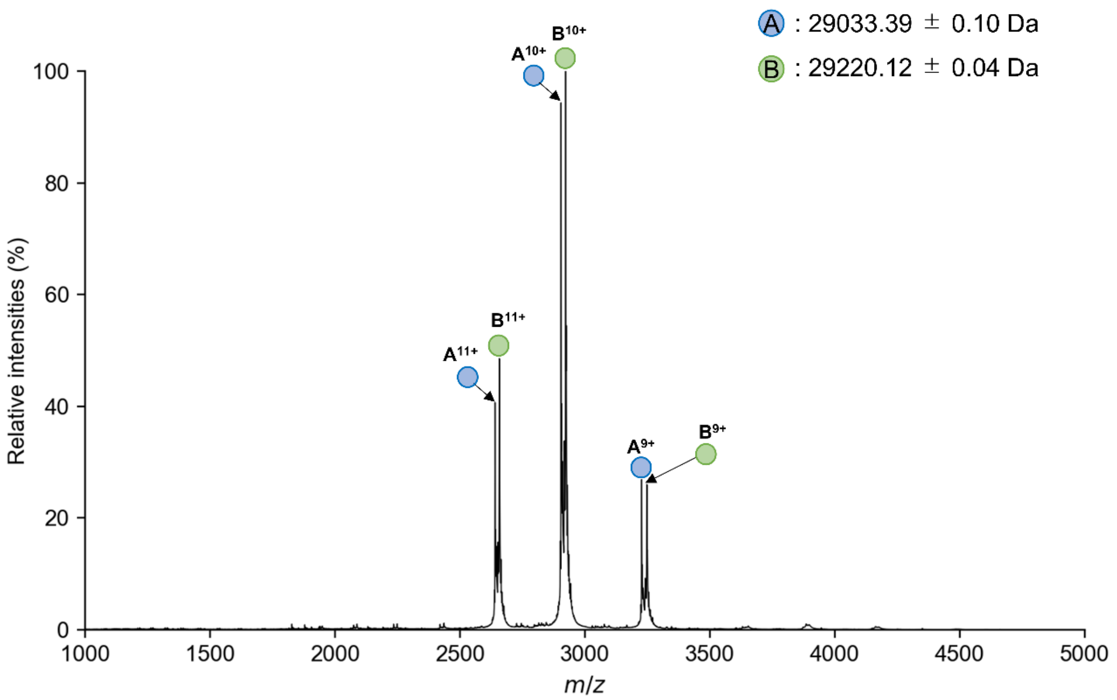

Figure S13. Stoichiometric analysis of the L78R mutant of SUMO-STAT3ND by native MS. Native MS spectrum of the L78R mutant of SUMO-STAT3ND (50 μM). The molecular mass of the SUMO-STAT3ND mutant is calculated to be 29,133.66 Da. A and B indicate a monomer of the SUMO-STAT3ND mutant.

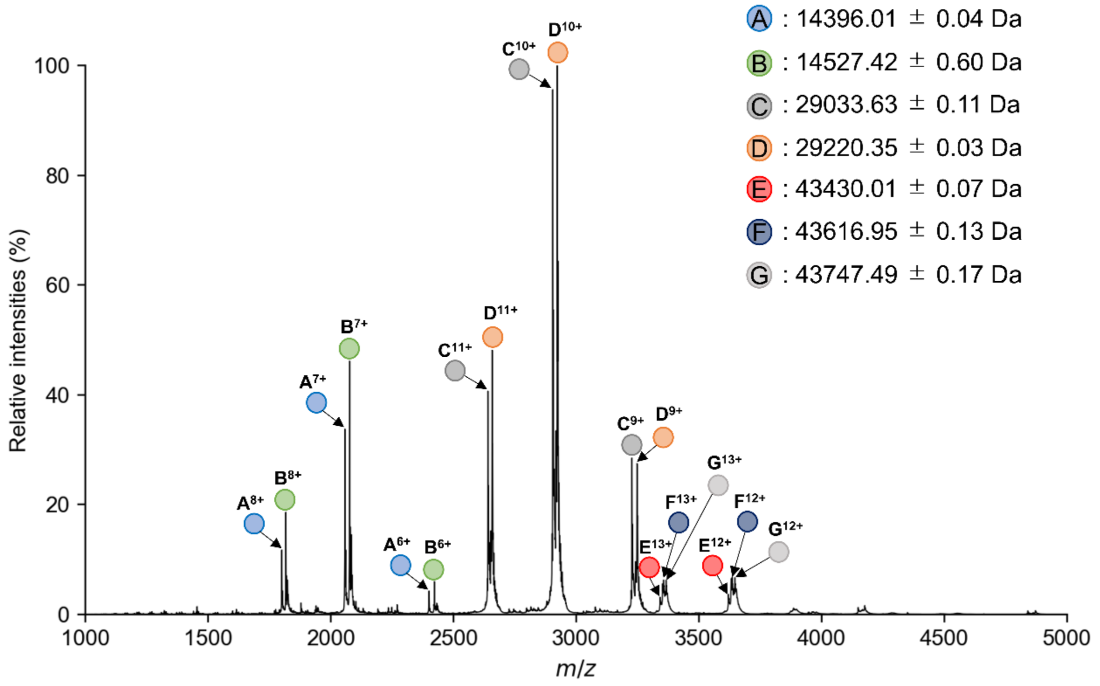

Figure S14. Stoichiometric analysis of the complex between Y3 and the L78R mutant of SUMO-STAT3ND by native MS. Native MS spectrum of the complex between Y3 and the L78R mutant of SUMO-STAT3ND (50 μM). The molecular mass of Y3 is calculated to be 14,526.92 Da, while that of the SUMO-STAT3ND mutant is calculated to be 29,133.66 Da. A indicates a monomer of Y3 lacking one amino acid residue, B indicates a monomer of Y3, C and D indicate a monomer of the SUMO-STAT3ND mutant, and E and F indicate a complex between Y3 and the L78R mutant of SUMO-STAT3ND at a ratio of 1:1.

Table S1. Summary of gBlocks used in this study.

Name	Sequence	Type
A DNA fragment encoding LgBiT	TCGAGCGGTGGTGGCGGGAGCGGAGGTGGAGGGTCGTCAGGTGTCTTCACACTCG<br>AAGATTTCGTTGGGGACTGGGAACAGACAGCCGCCTACAACCTGGACCAAGTCCT<br>TGAACAGGGAGGTGTGTCCAGTTTGCTGCAGAATCTCGCCGTGTCCGTAACTCCG<br>ATCCAAAGGATTGTCCGGAGCGGTGAAAATGCCCTGAAGATCGACATCCATGTCA<br>TCATCCCGTATGAAGGTCTGAGCGCCGACCAAATGGCCCAGATCGAAGAGGTGTT<br>TAAGGTGGTGTACCCTGTGGATGATCATCACTTTAAGGTGATCCTGCCCTATGGC<br>ACACTGGTAATCGACGGGGTTACGCCGAACATGCTGAACTATTTCGGACGGCCGT<br>ATGAAGGCATCGCCGTGTTCGACGGCAAAAAGATCACTGTAACAGGGACCCTGTG<br>GAACGGCAACAAAATTATCGACGAGCGCCTGATCACCCCCGACGGCTCCATGCTG<br>TTCCGAGTAACCATCAACAGTGGGAGTTCAGGAGGAGGTGGCAGCGGTGGAGGCG<br>GCTCGAGCTCATCAGGAGGAGGTGGTTCAGGTGGAGGTGGATCATCTGGAGTGAC<br>CGGCTACCGGCTGTTCGAGGAGATTCTGGGAAGCTCTGGAGGAGGTGGTAGCGGT<br>GGAGGCGGTTCAAGC	gBlocks (linear DNA)
A DNA fragment encoding SmBiT	CTCATCATTTTGGCAAAGAATTCGAGCTCATCGATGCATCTCGAGaccATGGTGA<br>CCGGCTACCGGCTGTTCGAGGAGATTCTCGGGAGTTCCGGTGGTGGCGGGAGCGG<br>TGGAGGTGGTACCAGT	gBlocks (linear DNA)

Supplementary Text S1. MS analysis using a mixed solution of Y3 and STAT3ND under denaturing condition with formic acid.

To determine the accurate molecular weights of subunits in the protein complex, native MS analysis was performed using a mixed solution of Y3 and STAT3ND under denaturing condition with formic acid. The spectrum showed peaks of molecular weights corresponding to monomeric Y3 and STAT3ND and those lacking one amino acid residue **(Fig. S7)**. In addition, a peak corresponding to the molecular weight of the Y3 dimer was observed **(Fig. S7)**. To confirm the dimerization of Y3, a heat-untreated Y3 sample was prepared and subjected to SDS-PAGE and western blot analysis. The results indicated that Y3 partially formed a homodimer **(Fig. S8)**. Y3 can form homodimers even under denaturing condition when its concentration is adequately high.

Supplementary Text S2. Binding analysis between Y3 and STAT3ND monomer.

The two *N*-terminal domains in a STAT1 homodimer are known to contribute to the activation-inactivation cycle via mutual interactions (14, 16, 17). However, when Leu78 of STAT3, which is located at the dimer interface between the *N*-terminal domains, is replaced by Arg, the formation of the latent dimer via the interaction between the *N*-terminal domains is inhibited (52). To investigate whether C protein binds to the monomeric form of STAT3ND, a binding analysis between Y3 and the L78R mutant of STAT3ND was conducted. In this case, the *N*-terminus of STAT3ND was fused to a small ubiquitin-related modifier (SUMO) tag to enhance its solubility and stability.

Control SEC analysis showed that SUMO-fused STAT3ND (SUMO-STAT3ND) alone eluted at approximately 14 mL, corresponding to 72 kDa, when the concentration was set at 15 μM **(Fig. S9*A*)**. Based on the calculated molecular mass of SUMO-STAT3ND (29 kDa), SUMO-STAT3ND is thought to form a dimer. When Y3 was added to the SUMO-STAT3ND solution, Y3 co-eluted with SUMO-STAT3ND at approximately 13.5 mL, corresponding to a molecular weight of 83 kDa, indicating that Y3 could bind to the SUMO-STAT3ND dimer **(Fig. S9*A*)**. In contrast, the L78R mutant of SUMO-STAT3ND alone was eluted at approximately 15 mL, corresponding to 43 kDa, suggesting that the mutant forms a monomer **(Fig. S9*B*)**. When a mixed solution

of Y3 and the SUMO-STAT3ND mutant was used, Y3 did not co-elute with the SUMO-STAT3ND mutant **(Fig. S9*B*)**. However, because the Y3 peak was broadened, Y3 was thought to partially interact with the STAT3ND monomer. These results indicate that Y3 binds to the dimeric form of STAT3ND rather than to its monomeric form.

To investigate whether Y3 could bind to the STAT3ND monomer, native MS analysis was conducted using SUMO-STAT3ND or the L78R mutant and Y3 **(Fig. S10)**. We found that although SUMO-STAT3ND alone forms a monomer and dimer, it also forms a heterotrimer with Y3 at a ratio of 2:1 **(Figs. S11 and S12)**. Furthermore, native MS analysis demonstrated that the L78R mutant of SUMO-STAT3ND alone forms a monomer, while the L78R mutant and Y3 can form a heterodimer **(Figs. S13 and S14)**. Although Y3 seems to weakly bind to STAT3ND monomer, Y3 and STAT3ND form a more stable heterotrimer at a ratio of 1:2.
